# Soluble ST2 does not change cardiovascular risk prediction compared to cardiac troponin T in kidney transplant candidates

**DOI:** 10.1371/journal.pone.0181123

**Published:** 2017-07-13

**Authors:** Mira T. Keddis, Ziad El-Zoghby, Bruce Kaplan, Jeffrey W. Meeusen, Leslie J. Donato, Fernando G. Cosio, D. Eric Steidley

**Affiliations:** 1 Division of Nephrology and Hypertension, Department of Medicine, Mayo Clinic, Phoenix, Arizona, United States of America; 2 Division of Nephrology and Hypertension, Department of Medicine, Mayo Clinic, Rochester, Minnesota, United States of America; 3 Division of Renal Laboratory, Department of Laboratory Medicine and Pathology, Mayo Clinic, Rochester, Minnesota, United States of America; 4 Division of Cardiology, Department of Medicine, Mayo Clinic, Phoenix, Arizona, United States of America; The University of Tokyo, JAPAN

## Abstract

**Background:**

Solubility of Tumorigenicity 2 (sST2) is a novel biomarker that better stratifies risk of cardiovascular events (CVE) compared to cardiac troponin T(cTnT) in heart failure. We assessed the association of sST2 with the composite outcome of CVE and/or mortality compared to cTnT in kidney transplant candidates.

**Methods:**

200 kidney transplant candidates between 2010 and 2013 were included. Elevated sST2 was defined as ≥30ng/ml, cTnT≥0.01 ng/ml.

**Results:**

Median age 53 (interquartile range (IQR) 42–61) years, 59.7% male and 82.0% white. 33.5% had history of CVE, 42.5% left ventricular hypertrophy (LVH) and 15.6% positive cardiac stress test. Elevated sST2 correlated with male gender, history of prior-transplants, CVE, positive stress test, LVH, elevated cTnT, anemia, hyperphosphatemia, increased CRP and non-transplanted status. Male gender, history of CVE and LVH were independent determinants of sST2. During 28 months (IQR 25.3–30), 7.5% died, 13.0% developed CVE and 19.0% developed the composite outcome. Elevated sST2 was associated with the composite outcome (hazard ratio = 1.76, CI 1.06–2.73, p = 0.029) on univariate analysis but not after adjusting for age, diabetes and cTnT (p = 0.068). sST2 did not change the risk prediction model for composite outcome after including age, diabetes, prior history of CVE and elevated cTnT.

**Conclusions:**

Increased sST2 level is significantly associated with variables associated with CVE in kidney transplant candidates. sST2 was associated with increased risk of the composite outcome of CVE and/or death but not independent of cTnT. Larger studies are needed to confirm these findings and determine whether sST2 has added value in CV risk stratification in this cohort of patients.

## Introduction

Assessment of cardiovascular risk (CV) in patients with advanced chronic kidney disease (CKD) presenting for kidney transplantation is an important component of every transplant evaluation; however the approach to CV assessment is largely transplant center specific. Besides a detailed history and physical examination, most centers include cardiac testing such as a structural evaluation, typically by echocardiography or coronary angiography and/or a functional assessment such as stress treadmill testing or myocardial perfusion imaging. Several biomarkers including N-terminal pro-B-type natriuretic peptide (NT-pro-BNP), high sensitivity C-reactive protein (hs-CRP) and cardiac troponin T (cTnT) have been studied and shown to correlate with adverse CV outcomes in the end-stage renal disease (ESRD) population[1–5]. Our group has previously shown that cTnT is a powerful predictor of CV mortality and morbidity in dialysis patients awaiting kidney transplant and post-transplant [2, 3, 6–9]. However, due to the effect of impaired glomerular filtration rate (GFR) on clearance of the cTnT molecule, understanding the clinical implications of changes in cTnT levels in advanced kidney disease has been challenging except for changes that occur after kidney transplantation [10–12].

Suppression of tumorigenicity 2 (ST2) is a novel biomarker of CV risk currently utilized for risk stratification and prognostication in patients with heart failure [13] as it provides better discrimination of CV morbidity and mortality compared to cTnT and NT-Pro-BNP [14–17]. Two studies evaluated the effect of GFR on sST2 levels and showed either no association or weak correlation with eGFR [18, 19]. To our knowledge, there are no reported studies on sST2 and ESRD patients. Therefore the objectives of this study are:1) to define the determinants of sST2; 2) its association with CV outcomes and mortality and 3) the discriminative value of sST2 compared to cTnT in predicting CV events and mortality in a cohort of patients with advanced CKD presenting for kidney transplant evaluation.

## Materials and methods

### Study population

The Mayo Clinic Institutional Review Board approved this study. Informed consent was waived. Outpatient adult patients with advanced CKD who presented for kidney transplant evaluation at Mayo Clinic, Rochester, MN between May 2010 and November 2013 were included. Patients with history of collagen vascular disease, rheumatoid arthritis, pulmonary fibrosis, chronic obstructive pulmonary disease and clinically significant asthma were excluded as levels of sST2 are known to be increased in these cohorts of patients.

### Laboratory and clinical assessment

Stored serum samples from all kidney transplant candidates between May 2010 and November 2013 were retrieved. Only samples with at least 75 microliters available for sST2 testing were included. sST2 was analyzed using a high-sensitivity sandwich immunoassay (Presage ST2; Critical Diagnostics, San Diego, CA). All samples with an elevated level were diluted to provide quantitative results. In patients with heart failure, the upper reference limit for sST2 is 35 nanograms per milliliter (ng/ml) [20, 21]. In this study, sST2 was analyzed as a continuous variable and as a binary variable with high sST2 defined as a value greater than or equal to 30 ng/ml. This cutoff value was considered based on a prior study of patients with systemic amyloidosis in which an upper reference limit of 30 ng/ml was shown to predict mortality [22]. cTnT was analyzed using the 4^th^ generation Roche Diagnostics assay and it was analyzed as a continuous variable and binary variable with an elevated cTnT defined as a value greater than or equal to 0.01 ng/ml which is the level above the point of detection. High sensitivity CRP was analyzed using the Roche assay and results were reported as low as <0.2 and levels above 2mg/L were considered abnormal. Serum albumin was also analyzed using Roche and HemoCue analyzer was used for hemoglobin.

Clinical characteristics were determined from the medical record including age, sex, race, height, weight, cause of CKD, diabetes status, number of prior transplants, dialysis, history of CV events, transplant committee decision and date of transplantation. Laboratory data abstracted at the time of the transplant evaluation included the following: serum levels of hemoglobin, albumin, uric acid, phosphorus, parathyroid hormone (PTH), creatinine, CRP and cTnT.

### Cardiovascular disease assessment

Cardiovascular disease assessment variables abstracted from chart review included echocardiogram findings of left ventricular hypertrophy (defined as left ventricular mass index (LVMI) greater than 103 g/m^2^ in men or LVMI greater than 89 g/m^2^ in women), left ventricular ejection fraction, and cardiac stress test findings. Cardiovascular events (CVE) were defined as: a history of acute myocardial infarction or coronary artery revascularization (surgical bypass grafting or percutaneous coronary intervention), history of hospitalization for heart failure, peripheral vascular disease defined as surgical or percutaneous lower extremity revascularization or limb amputation, central vascular disease including abdominal aortic aneurysm requiring surgical repair, carotid or renal artery stenosis requiring surgical or percutaneous intervention and cerebrovascular events defined as any ischemic or hemorrhagic cerebrovascular accident. These definitions applied for both pre and post-transplant CVE.

### Statistical analysis

Descriptive statistics such as number of observations, mean, median, standard deviation, and range were provided for quantitative variables, and qualitative or categorical variables were summarized using counts and percentages. Continuous data were reported as median with interquartile ranges (IQR). Wilcoxon Rank Sum test was used for comparison of continuous variables. Comparisons between more than two groups of data were performed by ANOVA for normally distributed and by Kruskall-Wallis test for skewed data. Proportions were compared by Chi-square. Survival analysis was performed using Cox proportional hazard models. Variables significantly associated with the composite outcome were identified by univariate analysis in which each variable was entered separately in the cox hazard model to determine its significance. Variables that were significantly correlated with each other were not entered in the final model together. The final multivariate cox hazard model was performed by entering all the significant variables at the same time. The significance level of the p-value was set at less than 0.05. All statistical analyses were performed using JMP, version 9 (SAS Institute, Cary, NC). The outcome of interest was the composite outcome of patient mortality and/or CVE. Follow-up was censored at the time of transplant or last follow-up or October 2015.

## Results

**Fig 1:**
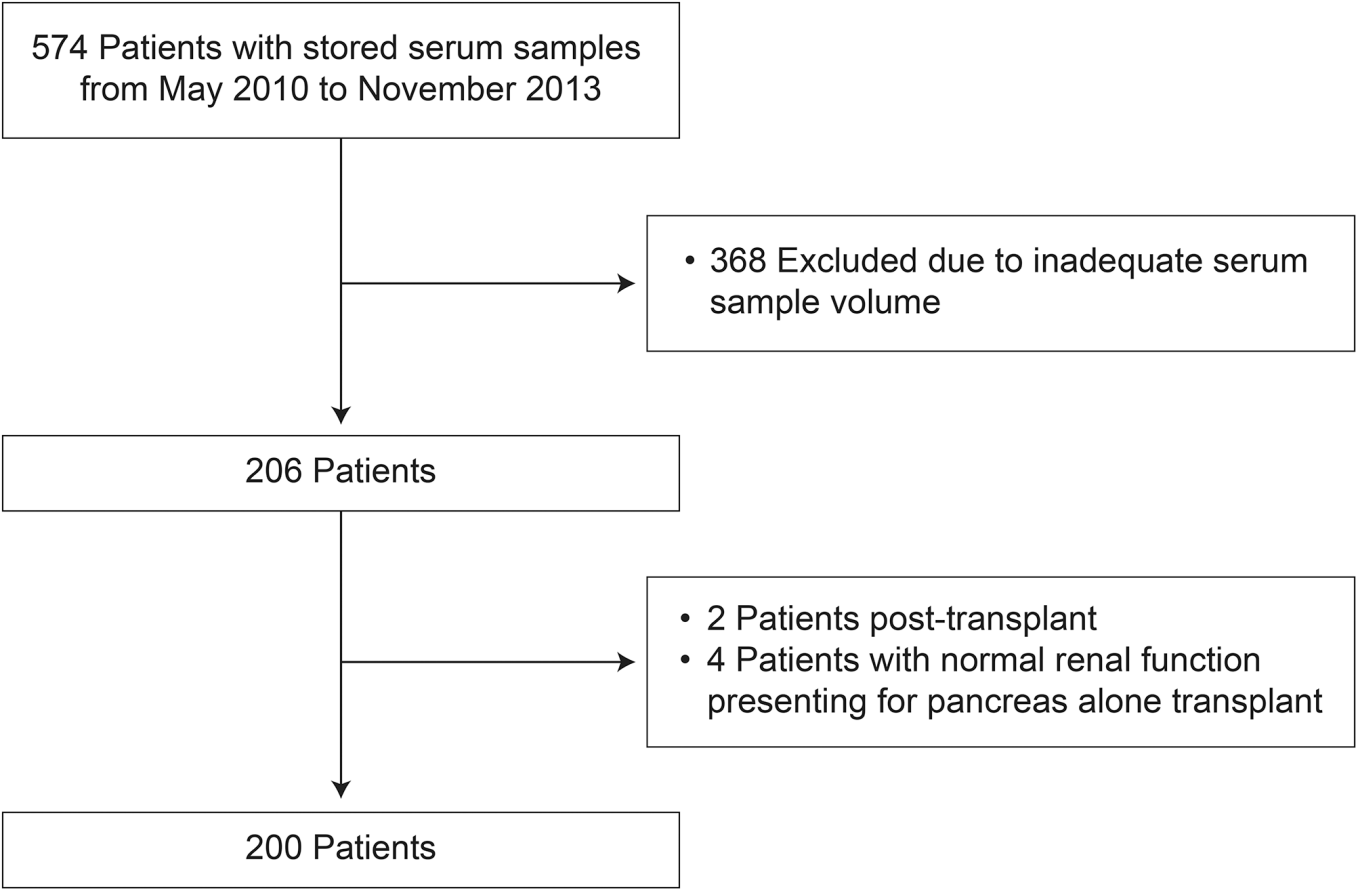
Flowchart of patients screened and included in the study.

Five hundred and seventy four patients evaluated at the transplant center from May 2010 to November 2013 had stored serum samples available for analysis, of whom 368 were excluded due to inadequate serum sample volume and 6 were excluded because of preserved kidney function as shown in Fig 1. None of the patients had disease processes that met our exclusion criteria.

Two hundred kidney transplant candidates were included in the study. This patient cohort was predominately white, male presenting for first kidney transplant evaluation as shown in Table 1. Diabetes was the predominant cause of CKD. Sixty percent of patients were on dialysis at the time of transplant evaluation for a median of 24 months. Sixty seven (33.5%) patients had a history of CVE. A dobutamine stress echocardiogram was obtained in 154 (77%) patients and was positive for ischemia in 24 (15.6%) patients. Left ventricular hypertrophy was noted in 92 (57.5%) patients and mean left ventricular ejection fraction was 61.1%. Median follow-up was 28 months after initial evaluation (IQR 25.3–30 months). One hundred and sixty six (83.4%) were approved for transplant and 84 (42.6%) were transplanted during the follow-up period; 96% of those transplanted had their procedure at Mayo Clinic Rochester.

**Table 1:** Baseline characteristics.

Patient characteristics	N = 200
**Recipient age–years**	**53 (42, 67)**
**Male gender–no. (%)**	**120 (59.7)**
**White race–no. (%)**	**164 (82.0)**
**Diabetes–no. (%)**	**77 (38.5)**
**Hypertension–no. (%)**	**189 (94.5)**
**Cause of end stage renal disease–no. (%)**	
**Diabetes:**	**54 (27.0)**
**Glomerular disease:**	**47 (23.5)**
**PCKD:**	**17 (8.5)**
**Other:**	**82 (41.0)**
**Re-transplants–no. (%)**	**39 (19.5)**
**Dialysis at the time of evaluation–no. (%)**	**119 (59.5)**
**Dialysis time (median months, interquartile range)**	**26 (11, 44.5)**
**History of cardiovascular events (CVE)–no. (%)**	**67 (33.5)**
**Left ventricular hypertrophy (LVH)–no. (%)**	**68 (42.5)**
**Positive stress echocardiogram–no. (%)**	**24 (15.6)**
**All-cause mortality–no. (%)**	**15 (7.5)**
**Composite outcome (CVE and/or death)**	**38 (19.0)**
**Transplant approval–no. (%)**	**166 (83.4)**
**Transplanted–no. (%)**	**84 (42.6)**
**Laboratory data**	**median (interquartile range)**
**Hemoglobin (g/dL)**	**11 (10.1, 11.9)**
**Serum albumin (g/dL)**	**4.2 (3.9, 4.4)**
**Uric acid (g/dL)**	**6.5 (5.2, 8.2)**
**Phosphorus (mg/dL)**	**4.8 (4.1, 5.6)**
**Parathyroid hormone**	**201.5 (107, 354)**
**Creatinine (mg/dL)**	**5.7 (4, 8.8)**
**C-reactive protein (mg/L)**	**2.7 (1.1, 7.4)**
**Cardiac troponin T (ng/mL)**	**0.02 (0.005, 0.05)**
**Soluble ST2 (ng/mL)**	**27.8 (19.3, 37.8)**

### Variables associated with sST2

**Fig 2:**
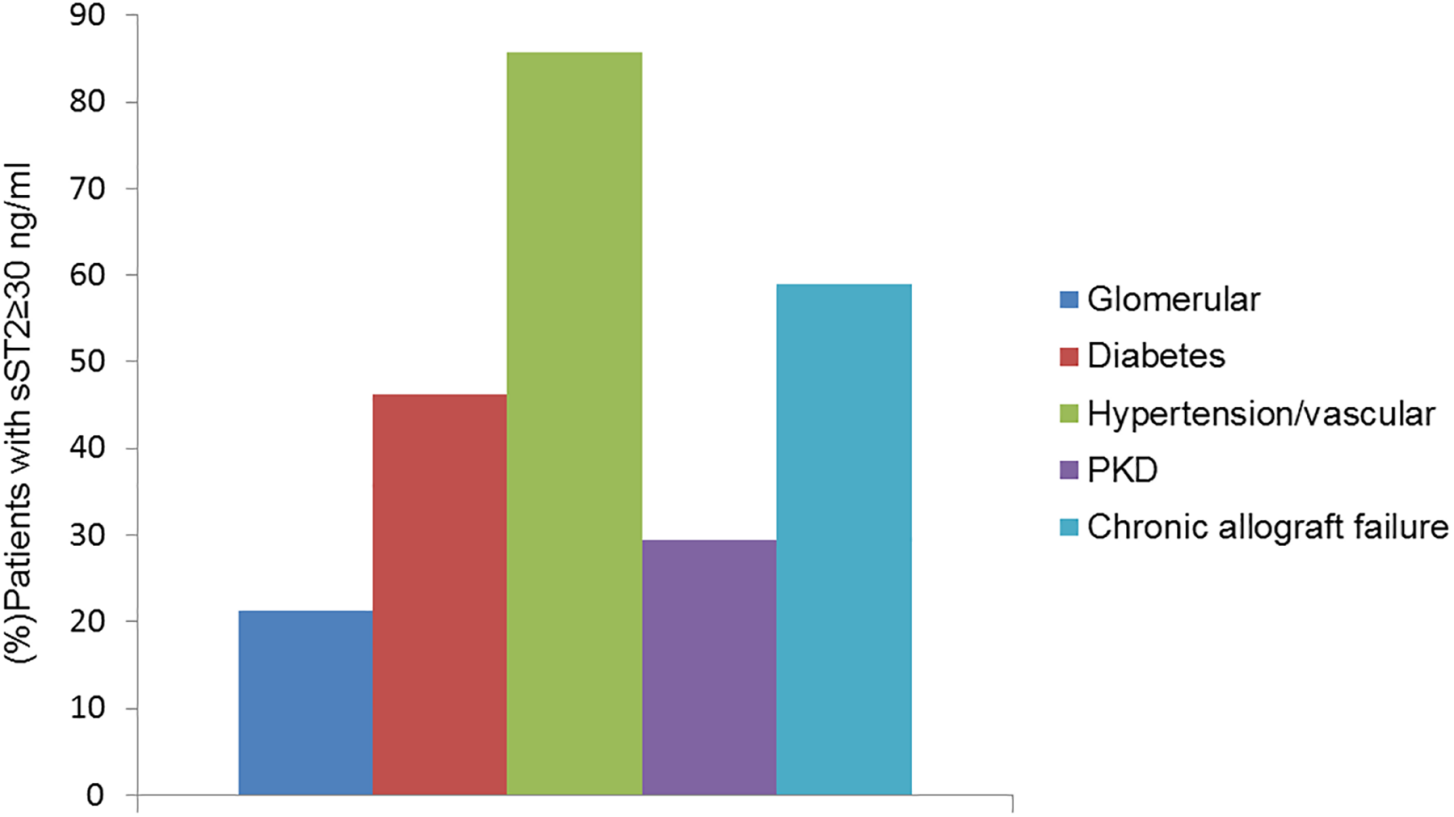
Distribution of patients with an elevated sST2 level across different causes of ESRD. sST2 level was higher in patients with ESRD secondary to hypertension, renovascular disease and chronic allograft failure compared to other causes.

The median sST2 in this cohort was 27.8 ng/ml (IQR 19.3–37.8), and 85 (42.5%) patients had an sST2 level ≥30ng/ml. The presence of an elevated sST2 level varied by the underlying cause of CKD. Patients with hypertension or renovascular etiology and patients with failed renal allograft were more likely to have an elevated sST2 level compared to patients with advanced CKD due to polycystic kidney disease or diabetes (p<0.001) **(Fig 2)**.

Next we evaluated clinical and laboratory variables associated with an elevated sST2 level. Patients with an elevated sST2 were more likely to be male and have a history of failed kidney transplant as shown in Table 2. An elevated sST2 was associated with several clinical variables of CV disease including history of CV events, positive stress echocardiogram and presence of LVH. sST2 strongly correlated with a positive cTnT (defined as a level ≥0.01ng/ml), anemia, hyperphosphatemia and an increased level of hsCRP. sST2 was not associated with age, race, diabetes, hypertension, dialysis status, and serum albumin levels (data not shown). On multivariate analysis, the following variables were independent determinants of an elevated sST2 level: male gender, history of CV events and presence of LVH as shown in Table 3.

**Table 2:** Clinical and laboratory variables associated with an elevated sST2 level.

Clinical and laboratory variables	sST2≥30ng/ml N = 85	sST2<30ng/ml N = 115	P value
**Male gender**	64 (53.3%)	56 (46.7%)	0.0001
**Retransplants**	23 (59.0%)	16 (41.0%)	0.02
**History of cardiovascular event**	39 (58.2%)	28 (41.8%)	0.001
**Positive stress echocardiogram**	15 (62.5%)	9 (37.5%)	0.041
**Left ventricular hypertrophy**	39 (57.4%)	29 (42.7%)	0.007
**Non-transplanted**	57 (50.4%)	56 (49.6%)	0.006
**cTnT>0.01ng/ml**	61 (54.0%)	52 (46.0%)	0.0002
**Hemoglobin (g/dL)**	10.7 (10.1, 11.6)	11.2 (10.2, 12)	0.049
**Phosphorus (mg/dL)**	5 (4.3, 6)	4.6 (4, 5.5)	0.043
**HsCRP (mg/L)**	3.3 (1.6, 10)	2.15 (0.8, 6.52)	0.004
**Cardiovascular event or Death**	22 (34.9%)	16 (13.9%)	0.033

**Table 3:** Univariate and multivariate analysis of determinants of an elevated sST2 level.

Clinical characteristics	Univariate		Multivariate	
	**OR (95% CI)**	**p-value**	**OR (95% CI)**	**p-value**
**Male gender**	3.21 (1.74, 5.93)	p = 0.0001	4.19 (2.03, 9.07)	p<0.0001
**Retransplants**	2.30 (1.13, 4.68)	p = 0.02	1.19 (0.49, 2.90)	p = 0.695
**cTnT>0.01ng/ml**	3.08 (1.69, 5.60)	p = 0.0002	-	
**History of cardiovascular event**	2.63 (1.44, 4.81)	p = 0.0014	2.48 (1.22, 5.15)	p = 0.012
**Left ventricular hypertrophy**	2.40 (1.26, 4.57)	p = 0.007	2.34 (1.13, 4.93)	p = 0.022

### Variables associated with cTnT

Median cTnT level was 0.02 ng/ml (IQR 0.005–0.05). One hundred and thirteen (56.5%) patients had an elevated cTnT level, of whom sixty one (54.0%) had an sST2≥ 30ng/ml. The following variables were associated with an elevated cTnT level: male gender (OR 2.61 (1.46, 4.70), p = 0.001), diabetes (OR 4.66 (2.49, 9.10), p<0.0001), dialysis (OR 3.57 (1.99, 6.53), p<0.0001), prior history of CVE (OR 4.61 (2.38, 9.37), p<0.0001), presence of LVH (OR 5.31 (2.24, 14.8), p<0.0001), positive cardiac stress test (OR 5.82 (1.89, 25.5), p = 0.006) and hyperphosphatemia (OR 4.71 (1.50, 16.1), p = 0.01).

### cTnT and sST2 levels and positive cardiac stress testing

Twenty-four patients of 154 (15.6%) had a stress echocardiogram positive for ischemia at the time of transplant evaluation. Twenty-one (87.5%) patients with positive test for ischemia had an elevated cTnT (p = 0.003) while 15 (62.5%) had an elevated sST2. Ten patients (41.7%) with positive stress echocardiogram required coronary angioplasty or coronary bypass surgery for severe coronary artery disease (CAD). Positive cardiac stress testing and an elevated cTnT were independent predictors of severe CAD requiring intervention [positive stress test: OR 3.38 (1.25, 9.05), p = 0.012, and cTnT: OR 2.98 (1.09, 9.53), p = 0.032). An elevated sST2 was not significantly associated with severe CAD requiring intervention independent of positive stress test and an elevated cTnT.

### cTnT and sST2 levels and the composite outcome of mortality and/or CV events after transplant evaluation

Over a median follow-up of 28 months (IQR 25.3–30), 15 (7.5%) died and 26 (13.0%) suffered CV events. Thirty eight (19.0%) suffered from the composite outcome of death and/or CV event. Twenty nine of the 38 patients (76.3%) suffered from the composite outcome while on the waitlist.

By univariate cox analysis (**Table 4**), several variables related to the composite endpoint including older age (age per 10 years HR 1.38 (1.07, 1.81), p = 0.0118), diabetes (HR 3.33 (1.70, 7.02), p = 0.0004), increased cTnT level (log cTnT HR 1.53 (1.23, 1.91), p = 0.0001), increased sST2 level (log sST2 HR 1.76 (1.06, 2.73), p = 0.029), prior history of CV events (HR 4.60 (2.34, 9.69), p<0.0001), positive stress echocardiogram (HR 3.07 (1.43, 6.26), p = 0.005), and an ejection fraction below 45% (HR 4.66 (1.57–11.1), p = 0.008). Neither serum creatinine (HR 1.34 (0.71, 2.57), p = 0.37) nor CRP (HR 1.01(0.78, 1.30), p = 0.95) was significantly associated with the composite outcome. On multivariate proportional hazard modeling we assessed the variables that were significantly associated with the composite outcome using two models. Model 1included age, diabetes and log cTnT levels. Model 2 included age, diabetes and log sST2 levels. In model 1, long transformed cTnT was significantly associated with the composite outcome after adjusting for age and diabetes. In model 2, sST2 was not a significant predictor of the composite outcome independent of age and diabetes. Therefore, only older age, increased level of cTnT and diabetes were independently associated with mortality and/or CV event during the follow-up time. An sST2 level ≥30ng/ml was not significantly associated with the composite outcome in univariate (HR 1.64 (0.86–3.17) or multivariate (HR 1.77 (0.92–3.48), p = 0.09) analysis. The relationship of an elevated cTnT with the composite outcome remained significant when cTnT was analyzed as a binary variable (≥0.01ng/ml) in univariate (HR 3.18 (1.48–7.86), p = 0.002) and multivariate (HR 2.23 (1.02–5.62), p = 0.045) analysis. In our cohort, event-free survival was not associated with gender or dialysis.

**Table 4:** Variables associated with the composite outcome of cardiovascular events and/or death.

Clinical characteristics	Univariate HR (CI)		Multivariate HR (CI)			
		p-value	*Model 1*	p-value	*Model 2*	p-value
**Age(per10 years)**	1.38(1.07, 1.81)	0.0118	1.34(1.03, 1.78)	p = 0.031	1.35(1.02, 1.81)	p = 0.004
**Diabetes**	3.33(1.70, 7.02)	0.0004	2.24(1.09, 4.89)	p = 0.027	2.88(1.46, 6.09)	p = 0.002
**Log cTnT**	1.53(1.23, 1.91)	0.0001	1.37(1.08, 1.74)	p = 0.008		
**Log sST2**	1.76(1.06, 2.73)	0.029			1.55(0.966, 2.34)	p = 0.068

In order to identify the incremental value of cTnT and sST2 in predicting the combined outcome of CVE and mortality, we identified the c-statistic of each biomarker when added to a model of well-established clinical CV risk factors. Logistic regression model of age, diabetes and history of CV events had a c-statistic of 0.79. The addition of cTnT as a continuous variable improved the c-statistic of the model to 0.82. The addition of sST2 as a continuous variable did not change the c-statistic of the model (0.82 → 0.82). S1 Data file includes data for this analysis.

## Discussion

The findings of this study show that an elevated sST2 correlated with variables predictive of CV events and mortality. Compared to cTnT, sST2 was not an independent predictor of the composite outcome and did not have an added incremental value to the risk prediction model based on clinical risk factors and cTnT.

Suppression of tumorigenicity 2 (ST2) belongs to the interleukin-1 receptor family with transmembrane (ST2L) and soluble (sST2) isoforms and functions as a danger signal in response to tissue damage, necrosis and death [23]. The soluble ST2 isoform functions as a decoy receptor for IL-33 and thus inhibits IL-33/ST2L intracellular signaling. IL-33 signaling is protective against cardiac fibrosis and hypertrophy. sST2 therefore blocks the protective effect of IL-33 and initiates a CD4 T-cell dependent inflammatory cascade. Soluble ST2 is released primarily from vascular endothelial cells and to a lesser extent by myocardial cells. It was recently approved by the FDA and advocated for by the ACCF/AHA guidelines for risk stratification and prognostication in patients with heart failure [13] due to its performance in predicting risk of sudden death, CV events, all-cause mortality and providing better discrimination of 2 year CV risk than NT-ProBNP and cTnT [14–17]. In addition to its very weak association with GFR, there are several unique characteristics of sST2: 1) low biological variability, 2) percentage change in sST2 levels are predictive of mortality in heart failure patients [24], and 3) sST2 levels decrease after implementation of cardioprotective pharmacologic interventions and the change in sST2 correlates with reduction in CV events [25, 26]. Because of the complexity of interpretation and utility of biomarker directed intervention in advanced CKD, sST2 is a much more lucrative biomarker than cTnT and NT-ProBNP for CV risk stratification particularly in kidney transplant candidates where evidence based CV assessment is lacking. Our study findings confirm the association of increased sST2 with CV risk factors. However compared to cTnT, sST2 did not provide added value in risk stratification. The association of sST2 with male gender, LVH and prior history of CV events mirrors the relationship of cTnT with these variables [8]. Unlike cTnT, sST2 did not correlate with age, diabetes, dialysis or time on dialysis, all well-known predictors of adverse CV outcomes on the wait-list and post-transplant [6, 7]. The association of sST2 with prior history of kidney transplantation was unexpected. Compared to patients with no prior history of kidney transplants, patients with history of kidney transplants had higher sST2 levels (32.9 (IQR 22.1–47.9) ng/ml vs 26.4 (IQR 18.9–34.5), p = 0.017). Whether this finding was due to chance or related to drug effect from immunosuppression or other factors remains speculative but further studies are needed to better understand this relationship.

It is worth noting that high sensitivity cTnT (hs cTnT) assay was not available at the time of this study. It has been shown that hs cTnT is elevated in 81% of patients with CKD not yet on dialysis and that elevations in hs cTnT are associated with adverse CV endpoints [27, 28]. As hs cTnT allows for detection of low levels of myocardial injury, we speculate that it would be more predictive of CV events and/or mortality compared to sST2 in kidney transplant candidates. Further studies are required to examine the role of hs cTnT in this population.

On univariate analysis, elevated sST2 was significantly associated with reduced event-free survival (HR = 1.76, CI (1.06, 2.73), p = 0.029), however this relationship was not independent of other known predictors of CV events and mortality such as older age, diabetes and an elevated cTnT. These findings may be explained by the following: first the median level of sST2 in this cohort was much lower than what has been reported in heart failure patients where sST2 cut-off of ≥35ng/ml is considered abnormal and a level >65ng/ml has the highest sensitivity and specificity in predicting mortality. In our cohort, the median sST2 level was 27.8 ng/ml, much lower than what has been reported in heart failure patients. Second, the association of sST2 with adverse outcome was significant when sST2 was analyzed as a continuous variable but not as a binary variable defined as ≥30ng/ml on univariate analysis. Third, sST2 was not affected by dialysis or diabetes, two important CV risk factors in this cohort, yet it was influenced by prior history of kidney transplants suggesting that sST2 may represent different pathophysiologic mechanisms in CKD.

There are several strengths of our study. To our knowledge, this is the first study of sST2 in a cohort of patients with advanced CKD uniformly evaluated for kidney transplant. The availability of pertinent clinical and laboratory variables including cTnT and hs-CRP allowed for a comprehensive analysis of sST2 determinants and follow-up extended to over two years from the day of evaluation. There are also several limitations that warrant discussion. First, because of the small sample size and the small number of events, a type II error cannot be excluded. Moreover, our patient cohort may not be representative of other cohorts of patients being evaluated for kidney transplant as only 60% required dialysis at the time of evaluation and the majority were white. Data on NT-proBNP was not available for comparison as it is not routinely performed as a standard test for patients in the outpatient setting during kidney transplant evaluation at our center. Lastly, data regarding timing of the blood draw with respect to dialysis is not known. This may impact the cTnT data as cTnT levels have been shown to increase after hemodialysis [29]. However sST2 levels are not known to be affected by dialysis [30].

## Conclusions

This study shows that in patients with advanced CKD evaluated for kidney transplant, sST2 is associated with several known CV risk factors. However the association of sST2 with increased risk of CV events and/ or mortality was not independent of older age, diabetes and an elevated cTnT level. Further studies are required to establish a reference range of significance in advanced CKD patients and better characterize the association of sST2 with CV outcomes.

## Supporting information

S1 Data Files for the data presented. (PDF)
