# Supplementary material for: Soluble ST2 does not change cardiovascular risk prediction compared to cardiac troponin T in kidney transplant candidates
Source: PLoS One. 2017 Jul 13;12(7):e0181123. doi: 10.1371/journal.pone.0181123 (PMC5509308; doi:10.1371/journal.pone.0181123)
Supplement: S1 Data — (PDF) [file pone.0181123.s001.pdf]

Gender	Caucasian (	AgeAtEval	age/10	logAge	logAge/SD	Dxgroup	dxgroups 1	Dxgroup (1
M	1	46	4.6	3.828641	13.00532	1	1	1
F	1	60	6	4.094345	13.90788	6	5	5
M	1	49	4.9	3.89182	13.21993	5	4	4
M	1	59	5.9	4.077537	13.85079	5	4	4
M	1	65	6.5	4.174387	14.17977	2	2	2
M	1	31	3.1	3.433987	11.66474	1	1	1
M	1	62	6.2	4.127134	14.01926	2	2	2
F	1	71	7.1	4.26268	14.47969	4	6	5
F	1	53	5.3	3.970292	13.48649	1	1	1
M	1	57	5.7	4.043051	13.73364	1	1	1
M	1	42	4.2	3.73767	12.69631	1	1	1
F	1	77	7.7	4.343805	14.75526	4	6	5
M	1	70	7	4.248495	14.43151	3	3	3
M	1	45	4.5	3.806662	12.93067	1	1	1
M	1	48	4.8	3.871201	13.14989	2	2	2
F	0	63	6.3	4.143135	14.07361	7	5	5
M	1	41	4.1	3.713572	12.61445	1	1	1
M	1	63	6.3	4.143135	14.07361	2	2	2
M	0	52	5.2	3.951244	13.42179	2	2	2
F	0	37	3.7	3.610918	12.26575	1	1	1
M	1	63	6.3	4.143135	14.07361	1	1	1
M	1	56	5.6	4.025352	13.67352	5	4	4
F	1	39	3.9	3.663562	12.44457	5	4	4
F	1	61	6.1	4.110874	13.96403	3	3	3
M	1	50	5	3.912023	13.28856	1	1	1
M	1	58	5.8	4.060443	13.79272	2	2	2
F	1	47	4.7	3.850148	13.07838	2	2	2
F	1	26	2.6	3.258097	11.06727	1	1	1
M	1	67	6.7	4.204693	14.28271	1	1	1
F	1	48	4.8	3.871201	13.14989	6	5	5
F	1	27	2.7	3.295837	11.19547	7	5	5
M	1	70	7	4.248495	14.43151	4	6	5
F	1	60	6	4.094345	13.90788	5	4	4
M	0	57	5.7	4.043051	13.73364	1	1	1
F	1	27	2.7	3.295837	11.19547	1	1	1
M	0	42	4.2	3.73767	12.69631	5	4	4
F	1	74	7.4	4.304065	14.62027	5	4	4
M	1	67	6.7	4.204693	14.28271	1	1	1
F	1	53	5.3	3.970292	13.48649	1	1	1
M	1	67	6.7	4.204693	14.28271	5	4	4
M	1	61	6.1	4.110874	13.96403	4	6	5
F	1	59	5.9	4.077537	13.85079	3	3	3
M	0	53	5.3	3.970292	13.48649	2	2	2
F	1	40	4	3.688879	12.53057	5	4	4
M	1	50	5	3.912023	13.28856	5	4	4
M	1	54	5.4	3.988984	13.54998	6	5	5

F	0	62	6.2	4.127134	14.01926	1	1	1
M	0	50	5	3.912023	13.28856	1	1	1
F	0	42	4.2	3.73767	12.69631	3	3	3
M	1	22	2.2	3.091042	10.49981	5	4	4
F	1	71	7.1	4.26268	14.47969	2	2	2
F	1	57	5.7	4.043051	13.73364	3	3	3
M	1	54	5.4	3.988984	13.54998	7	5	5
M	1	64	6.4	4.158883	14.12711	1	1	1
M	1	51	5.1	3.931826	13.35583	5	4	4
F	1	65	6.5	4.174387	14.17977	2	2	2
M	1	48	4.8	3.871201	13.14989	1	1	1
F	1	62	6.2	4.127134	14.01926	7	5	5
M	0	63	6.3	4.143135	14.07361	2	2	2
M	0	42	4.2	3.73767	12.69631	5	4	4
M	1	71	7.1	4.26268	14.47969	7	5	5
F	1	52	5.2	3.951244	13.42179	2	2	2
M	1	70	7	4.248495	14.43151	3	3	3
M	0	45	4.5	3.806662	12.93067	1	1	1
M	1	34	3.4	3.526361	11.97852	2	2	2
M	0	28	2.8	3.332205	11.319	5	4	4
M	0	42	4.2	3.73767	12.69631	2	2	2
M	1	39	3.9	3.663562	12.44457	5	4	4
M	1	51	5.1	3.931826	13.35583	5	4	4
F	1	59	5.9	4.077537	13.85079	2	2	2
F	1	41	4.1	3.713572	12.61445	7	5	5
M	1	45	4.5	3.806662	12.93067	2	2	2
M	1	65	6.5	4.174387	14.17977	2	2	2
M	1	56	5.6	4.025352	13.67352	2	2	2
M	1	40	4	3.688879	12.53057	3	3	3
M	1	46	4.6	3.828641	13.00532	3	3	3
F	1	38	3.8	3.637586	12.35634	1	1	1
M	0	40	4	3.688879	12.53057	1	1	1
M	1	45	4.5	3.806662	12.93067	1	1	1
M	1	38	3.8	3.637586	12.35634	2	2	2
M	1	28	2.8	3.332205	11.319	5	4	4
F	0	25	2.5	3.218876	10.93404	1	1	1
M	1	44	4.4	3.78419	12.85433	2	2	2
F	1	63	6.3	4.143135	14.07361	2	2	2
F	1	24	2.4	3.178054	10.79538	1	1	1
M	1	43	4.3	3.7612	12.77624	7	5	5
F	1	46	4.6	3.828641	13.00532	1	1	1
M	1	60	6	4.094345	13.90788	2	2	2
M	1	33	3.3	3.496508	11.87712	2	2	2
F	1	46	4.6	3.828641	13.00532	2	2	2
M	0	44	4.4	3.78419	12.85433	2	2	2
M	1	43	4.3	3.7612	12.77624	2	2	2
M	1	65	6.5	4.174387	14.17977	7	5	5

F	1	49	4.9	3.89182	13.21993	2	2	2
M	1	45	4.5	3.806662	12.93067	2	2	2
F	1	60	6	4.094345	13.90788	7	5	5
F	0	34	3.4	3.526361	11.97852	1	1	1
M	1	68	6.8	4.219508	14.33304	1	1	1
F	1	75	7.5	4.317488	14.66586	7	5	5
M	1	63	6.3	4.143135	14.07361	2	2	2
F	1	58	5.8	4.060443	13.79272	4	6	5
M	1	61	6.1	4.110874	13.96403	7	5	5
M	1	49	4.9	3.89182	13.21993	5	4	4
M	1	46	4.6	3.828641	13.00532	7	5	5
M	0	52	5.2	3.951244	13.42179	7	5	5
M	1	17	1.7	2.833213	9.624004	5	4	4
M	1	61	6.1	4.110874	13.96403	1	1	1
F	1	57	5.7	4.043051	13.73364	5	4	4
M	0	42	4.2	3.73767	12.69631	2	2	2
F	1	38	3.8	3.637586	12.35634	6	5	5
F	1	57	5.7	4.043051	13.73364	2	2	2
M	0	34	3.4	3.526361	11.97852	2	2	2
F	1	57	5.7	4.043051	13.73364	2	2	2
M	0	33	3.3	3.496508	11.87712	2	2	2
F	0	31	3.1	3.433987	11.66474	2	2	2
M	1	70	7	4.248495	14.43151	1	1	1
F	0	61	6.1	4.110874	13.96403	2	2	2
M	1	44	4.4	3.78419	12.85433	7	5	5
F	1	73	7.3	4.290459	14.57405	2	2	2
M	1	49	4.9	3.89182	13.21993	5	4	4
M	1	41	4.1	3.713572	12.61445	3	3	3
M	1	69	6.9	4.234107	14.38263	7	5	5
F	1	55	5.5	4.007333	13.61231	3	3	3
M	1	46	4.6	3.828641	13.00532	4	6	5
F	0	64	6.4	4.158883	14.12711	2	2	2
M	1	57	5.7	4.043051	13.73364	1	1	1
M	1	62	6.2	4.127134	14.01926	3	3	3
F	1	20	2	2.995732	10.17606	1	1	1
M	1	54	5.4	3.988984	13.54998	3	3	3
M	1	58	5.8	4.060443	13.79272	7	5	5
M	1	61	6.1	4.110874	13.96403	3	3	3
F	1	50	5	3.912023	13.28856	2	2	2
M	1	51	5.1	3.931826	13.35583	5	4	4
F	1	72	7.2	4.276666	14.5272	7	5	5
F	1	30	3	3.401197	11.55336	1	1	1
F	1	29	2.9	3.367296	11.4382	1	1	1
F	1	56	5.6	4.025352	13.67352	2	2	2
M	1	49	4.9	3.89182	13.21993	2	2	2
M	1	54	5.4	3.988984	13.54998	7	5	5
M	1	27	2.7	3.295837	11.19547	5	4	4

F	0	21	2.1	3.044522	10.34179	1	1	1
F	1	58	5.8	4.060443	13.79272	2	2	2
F	1	64	6.4	4.158883	14.12711	2	2	2
M	1	64	6.4	4.158883	14.12711	7	5	5
F	1	56	5.6	4.025352	13.67352	6	5	5
M	1	56	5.6	4.025352	13.67352	5	4	4
F	1	27	2.7	3.295837	11.19547	5	4	4
F	1	58	5.8	4.060443	13.79272	1	1	1
F	1	69	6.9	4.234107	14.38263	7	5	5
M	1	48	4.8	3.871201	13.14989	5	4	4
M	1	43	4.3	3.7612	12.77624	1	1	1
M	1	39	3.9	3.663562	12.44457	1	1	1
F	1	42	4.2	3.73767	12.69631	5	4	4
M	1	50	5	3.912023	13.28856	2	2	2
F	1	68	6.8	4.219508	14.33304	3	3	3
M	1	58	5.8	4.060443	13.79272	5	4	4
F	1	57	5.7	4.043051	13.73364	7	5	5
M	1	55	5.5	4.007333	13.61231	2	2	2
F	1	48	4.8	3.871201	13.14989	1	1	1
M	1	47	4.7	3.850148	13.07838	1	1	1
F	1	28	2.8	3.332205	11.319	2	2	2
F	1	31	3.1	3.433987	11.66474	5	4	4
M	1	45	4.5	3.806662	12.93067	2	2	2
M	1	59	5.9	4.077537	13.85079	7	5	5
M	1	65	6.5	4.174387	14.17977	2	2	2
M	1	57	5.7	4.043051	13.73364	1	1	1
M	1	45	4.5	3.806662	12.93067	5	4	4
M	1	40	4	3.688879	12.53057	7	5	5
F	1	61	6.1	4.110874	13.96403	7	5	5
F	1	56	5.6	4.025352	13.67352	2	2	2
M	1	54	5.4	3.988984	13.54998	2	2	2
M	1	40	4	3.688879	12.53057	5	4	4
F	1	51	5.1	3.931826	13.35583	7	5	5
F	1	29	2.9	3.367296	11.4382	1	1	1
F	1	70	7	4.248495	14.43151	2	2	2
M	0	64	6.4	4.158883	14.12711	3	3	3
M	1	37	3.7	3.610918	12.26575	5	4	4
M	1	68	6.8	4.219508	14.33304	2	2	2
M	1	45	4.5	3.806662	12.93067	5	4	4
F	0	56	5.6	4.025352	13.67352	4	6	5
F	0	65	6.5	4.174387	14.17977	7	5	5
F	0	65	6.5	4.174387	14.17977	7	5	5
F	0	62	6.2	4.127134	14.01926	2	2	2
F	1	63	6.3	4.143135	14.07361	3	3	3
M	1	56	5.6	4.025352	13.67352	5	4	4
F	1	52	5.2	3.951244	13.42179	5	4	4
M	0	27	2.7	3.295837	11.19547	1	1	1

M	1	63	6.3	4.143135	14.07361	1	1	1
F	1	57	5.7	4.043051	13.73364	7	5	5
M	1	55	5.5	4.007333	13.61231	7	5	5
F	0	48	4.8	3.871201	13.14989	1	1	1
M	1	25	2.5	3.218876	10.93404	1	1	1
M	0	65	6.5	4.174387	14.17977	2	2	2
M	1	50	5	3.912023	13.28856	7	5	5
M	1	58	5.8	4.060443	13.79272	3	3	3
F	0	58	5.8	4.060443	13.79272	7	5	5
M	1	51	5.1	3.931826	13.35583	5	4	4
M	1	64	6.4	4.158883	14.12711	5	4	4
M	0	77	7.7	4.343805	14.75526	5	4	4
M	1	40	4	3.688879	12.53057	5	4	4

Retranspla	HTN	Diabetes	Dialysis tim	log dialysis	Dialysis_At	Hgb	logHgb	Alb
0	y	n	2	0.693147	1	10.9	2.388763	4.1
0	y	n	48	3.871201	1	10	2.302585	4.6
1	y	n	26	3.258097	1	10.6	2.360854	4.6
1	y	n	45	3.806662	1	11.4	2.433613	3.5
0	y	y	37	3.610918	1	11.4	2.433613	4.1
0	y	n			0	11.6	2.451005	4.5
0	y	y	3	1.098612	1	10.4	2.341806	4.5
0	y	n			0	12	2.484907	4.2
0	n	n			0	9.4	2.24071	4.6
0	y	n	41	3.713572	1	12.1	2.493205	3.9
0	y	n	19	2.944439	1	11.3	2.424803	3.6
0	y	n	67	4.204693	1	11.6	2.451005	4.2
0	y	n			0	12.5	2.525729	4.2
0	y	n	16	2.772589	1	12.6	2.533697	4.2
0	y	y	69	4.234107	1	12.1	2.493205	3.5
0	y	y			0	11.3	2.424803	4.7
0	y	n	26	3.258097	1	10	2.302585	5
0	y	y	48	3.871201	1	12.1	2.493205	4.3
0	y	y	20	2.995732	1	9.5	2.251292	4
0	y	n	80	4.382027	1	12.1	2.493205	4.6
0	y	n	25	3.218876	1	12.1	2.493205	4.4
1	y	n	99	4.59512	1	9.6	2.261763	3.9
1	y	n	25	3.218876	1	12.3	2.509599	4.6
0	y	n	52	3.951244	1	11.3	2.424803	3.8
0	y	n	54	3.988984	1	10.7	2.370244	4.6
0	y	y	23	3.135494	1	11	2.397895	4.7
0	n	y	15	2.70805	1	12	2.484907	4.3
0	y	n	71	4.26268	1	10.7	2.370244	5
0	y	n	11	2.397895	1	11.5	2.442347	4.5
0	y	y	50	3.912023	1	11.3	2.424803	4.3
0	y	n	119	4.779123	1	9.2	2.219203	3.7
0	y	y	20	2.995732	1	10	2.302585	4.3
1	y	n	47	3.850148	1	11.7	2.459589	4.4
0	y	n			0	10.6	2.360854	2.6
0	y	n	31	3.433987	1	9.3	2.230014	4
1	y	n	9	2.197225	1	10.8	2.379546	3.9
1	y	n	61	4.110874	1	11.5	2.442347	4
0	y	n	42	3.73767	1	10.9	2.388763	4.4
0	y	n	28	3.332205	1	12.4	2.517696	4.3
1	y	n	64	4.158883	1	12.1	2.493205	3.8
0	y	n	44	3.78419	1	9.8	2.282382	4.6
0	y	n			0	10.9	2.388763	4.2
0	y	y	16	2.772589	1	10.9	2.388763	4.3
1	y	n	148	4.997212	1	9	2.197225	4.2
1	y	y	37	3.610918	1	12.2	2.501436	4.1
0	y	n	28	3.332205	1	11.2	2.415914	4.2

0	y	y			0	11.4	2.433613	4
0	y	n	30	3.401197	1	11.7	2.459589	4.1
0	y	n	38	3.637586	1	10.5	2.351375	4.7
1	y	n	18	2.890372	1	10.8	2.379546	4.7
0	y	y	35	3.555348	1	11.3	2.424803	4.4
0	y	n			0	9.4	2.24071	3.9
0	y	n	91	4.51086	1	11.2	2.415914	4.4
0	y	n	3	1.098612	1	12.4	2.517696	3.9
1	y	n			0	12.5	2.525729	4.2
0	y	y	22	3.091042	1	8.5	2.140066	3.1
0	y	n	61	4.110874	1	10.5	2.351375	4.2
0	y	y	8	2.079442	1	10.8	2.379546	4.1
0	y	y	26	3.258097	1	10.1	2.312535	4.6
1	y	n	26	3.258097	1	9.4	2.24071	3.9
0	y	n			0	9.6	2.261763	4.2
0	y	y			0	12.8	2.549445	4.4
0	y	y			0	12.4	2.517696	4
0	y	n			0	11	2.397895	3.9
0	y	y			0	11	2.397895	3.9
1	y	n	19	2.944439	1	11	2.397895	4.7
0	y	y	20	2.995732	1	10.2	2.322388	4.6
1	y	n	26	3.258097	1	11.6	2.451005	4.5
1	y	y			0	8.6	2.151762	3.8
0	y	y			0	12.4	2.517696	4.3
0	y	n			0	12.3	2.509599	4
0	y	y			0	8.8	2.174752	
0	y	y			0	11.7	2.459589	4.1
0	y	y	5	1.609438	1	11.8	2.4681	4.8
0	y	n			0	12.1	2.493205	4.3
0	y	n			0	10.2	2.322388	4.2
0	y	n	31	3.433987	1	12.3	2.509599	4
0	y	n	50	3.912023	1	11.3	2.424803	4.7
0	y	n			0	11.1	2.406945	4.9
0	y	y	7	1.94591	1	10.8	2.379546	4.4
1	y	n	134	4.89784	1	12.3	2.509599	4.5
0	y	n			0	10.2	2.322388	3.8
0	y	y	11	2.397895	1	10.5	2.351375	4.2
0	y	y	12	2.484907	1	10.7	2.370244	4.1
0	y	n	1	0	1	8.7	2.163323	4
0	n	y	5	1.609438	1	11.3	2.424803	3.7
0	y	n	3	1.098612	1	10.4	2.341806	3.2
0	y	y			0	12.3	2.509599	4.5
0	y	y	33	3.496508	1	10.1	2.312535	4.2
0	n	y	38	3.637586	1	8.7	2.163323	3.5
0	y	y	30	3.401197	1	10.2	2.322388	3.1
0	y	y			0	10.1	2.312535	4.1
0	y	y			0	10.1	2.312535	3.8

0	y	y	99	4.59512	1	12.3	2.509599	4.3
0	y	y	31	3.433987	1	12	2.484907	4.7
0	y	y			0	11	2.397895	4.4
0	y	n			0	9.7	2.272126	3.5
0	y	n			0	11.8	2.4681	4.3
0	y	n			0	11	2.397895	4.5
0	y	y			0	10.5	2.351375	3.6
0	y	n	28	3.332205	1	11.3	2.424803	4.9
0	n	n			0	14.9	2.701361	4
1	y	n			1	9.1	2.208274	3.7
0	n	n	12	2.484907	1	12.3	2.509599	3.7
0	y	n			0	8.3	2.116256	3.7
1	y	n			0	10	2.302585	4.9
0	y	n			0	13.8	2.624669	4.3
1	n	y			0	10.3	2.332144	4.2
0	y	y	10	2.302585	1	12	2.484907	4.3
0	y	n			1	9.5	2.251292	3.4
0	y	y	17	2.833213	1	12	2.484907	4.1
0	y	y			0	8.5	2.140066	3.8
0	y	y			0	11	2.397895	4.4
0	y	y			0	10.4	2.341806	4.4
0	y	y	18	2.890372	1	10.6	2.360854	4.7
0	y	n			0	9.6	2.261763	4.1
0	y	y			0	10.2	2.322388	4.1
0	y	n	20	2.995732	1	11.6	2.451005	3.8
0	y	y			0	10.9	2.388763	4.2
1	y	n	2	0.693147	1	10.9	2.388763	3.9
0	y	n			0	11.3	2.424803	4.9
0	y	n	32	3.465736	1	11.3	2.424803	4.4
0	y	n			0	10.4	2.341806	4.2
0	y	y	12	2.484907	1	11.8	2.4681	3.9
0	y	y	61	4.110874	1	10.5	2.351375	3.7
0	y	n			0	12.2	2.501436	4.2
0	y	n			0	13	2.564949	4
0	y	n	1	0	1	10	2.302585	3.4
0	y	n	25	3.218876	1	12.8	2.549445	4.5
0	y	n	4	1.386294	1	11.9	2.476538	4.9
0	y	n			0	10.3	2.332144	4.3
0	y	y			0	10.5	2.351375	3
1	y	n	8	2.079442	1	10.4	2.341806	4.3
0	y	n			0	11.3	2.424803	4.1
0	y	n	4	1.386294	1	11.6	2.451005	4.7
0	y	n	49	3.89182	1	14.6	2.681022	3.5
0	y	y	22	3.091042	1	12.9	2.557227	4.1
0	y	y			0	9.9	2.292535	4.1
0	y	n			0	10.7	2.370244	4.1
1	y	n	7	1.94591	1	12.3	2.509599	4.4

0	y	n			0	11	2.397895	4
0	y	y			0	10.9	2.388763	4
0	y	y			0	10.3	2.332144	3.4
0	y	y			0	10.3	2.332144	4
0	y	n	4	1.386294	1	12	2.484907	4.3
1	y	n			0	8	2.079442	3
1	y	n			0	8.9	2.186051	3.7
0	y	n			0	10.7	2.370244	4.1
0	y	n	19	2.944439	1	9.6	2.261763	3.4
1	y	y	4	1.386294	1	10.1	2.312535	3.4
0	y	n			0	10.8	2.379546	4.3
0	y	n			0	10.6	2.360854	3.6
1	y	y	39	3.663562	1	11.5	2.442347	4.7
0	y	y	49	3.89182	1	13.6	2.61007	4.1
0	y	n			0	10.7	2.370244	4.4
1	y	y	106	4.663439	1	10.1	2.312535	3.6
0	y	y			0	10.2	2.322388	4.2
0	y	y	4	1.386294	1	11.9	2.476538	3.9
0	y	n	22	3.091042	1	11.1	2.406945	4.1
0	y	n			0	11.5	2.442347	3.7
0	y	y			0	8.8	2.174752	4.1
1	n	n	8	2.079442	1	12.4	2.517696	4.3
0	y	y			0	12.4	2.517696	3.8
0	n	n	23	3.135494	1	11.5	2.442347	4.2
0	y	y			0	9.2	2.219203	4.2
0	y	n			0	13.5	2.60269	3.9
1	y	n			0	9	2.197225	3.8
0	n	n	3	1.098612	1	11.6	2.451005	3.1
0	y	n	51	3.931826	1	9.1	2.208274	5
0	y	y	21	3.044522	1	9.7	2.272126	3.4
0	y	y	8	2.079442	1	11.5	2.442347	4.3
1	y	y	36	3.583519	1	10.9	2.388763	4.4
0	y	y			0	8.1	2.091864	3.5
0	y	n	5	1.609438	1	12	2.484907	3.5
0	y	y			0	8.4	2.128232	3.8
0	y	y			0	12	2.484907	4.4
1	y	n	22	3.091042	1	10.1	2.312535	4.1
0	y	y			0	9.6	2.261763	4
1	y	n			0	10.6	2.360854	3.6
0	y	n			0	10.6	2.360854	3.5
0	y	y	13	2.564949	1	12.2	2.501436	3.9
0	y	n	1	0	1	9.9	2.292535	3.4
0	y	y			0	9.6	2.261763	2.9
0	y	n	2	0.693147	1	11.2	2.415914	4.3
1	y	n	57	4.043051	1	11.6	2.451005	4.6
1	y	y	49	3.89182	1	11	2.397895	4.2
0	y	n	7	1.94591	1	12.2	2.501436	4.9

0	y	n			0	11	2.397895	4.3
0	n	n	19	2.944439	1	9.3	2.230014	3.6
0	y	n			0	12.5	2.525729	4.2
0	y	n	31	3.433987	1	11.6	2.451005	4.3
0	y	n	30	3.401197	1	11.8	2.4681	5.1
0	y	y	39	3.663562	1	9.9	2.292535	4.7
0	y	n	73	4.290459	1	12.6	2.533697	4.4
0	y	n			0	12.6	2.533697	4.3
0	y	n	40	3.688879	1	10.3	2.332144	3.9
1	y	n			0	8.1	2.091864	4.1
1	y	n	35	3.555348	1	10.2	2.322388	4
1	y	n			0	9.6	2.261763	3.8
1	y	n	4	1.386294	1	11.6	2.451005	4.1

logAlb	Creat_TA	Uric acid	logUric aac	phosphoru	log Phosph	C-RP	CRP>3.0	log CRP
1.410987	10.5	8.3	2.116256	6.6	1.88707	11.1	1	2.406945
1.526056	5.3	3.2	1.163151	3.4	1.223775	1.5	1	0.405465
1.526056	9.5	3.9	1.360977	6.3	1.84055	5.6	1	1.722767
1.252763	14.2	6.2	1.824549	5	1.609438	2.7	1	0.993252
1.410987	8	7.7	2.04122	6.2	1.824549	5.7	1	1.740466
1.504077	3.8	7.7	2.04122	5.5	1.704748	0.4	1	-0.91629
1.504077	6.5	3.8	1.335001	5.4	1.686399	3.5	1	1.252763
1.435085	1.7	5.5	1.704748	4	1.386294	1.9	1	0.641854
1.526056	6.5	8.4	2.128232	5.2	1.648659	3.3	1	1.193922
1.360977	11.6	5.3	1.667707	3.5	1.252763	11	1	2.397895
1.280934	11.8	5.8	1.757858	4.7	1.547563	1.7	1	0.530628
1.435085	6.5	5.4	1.686399	8.1	2.091864	4.2	1	1.435085
1.435085	3.6	7.9	2.066863	4.6	1.526056	1.2	1	0.182322
1.435085	7.9	3.4	1.223775	5	1.609438	3	1	1.098612
1.252763	9.4	5.1	1.629241	6.6	1.88707	4.6	1	1.526056
1.547563	4.4	8.7	2.163323	4.8	1.568616	3.5	1	1.252763
1.609438	12.2	6.7	1.902108	7.2	1.974081	0.7	1	-0.35667
1.458615	6.6	5.6	1.722767	4.8	1.568616	8	1	2.079442
1.386294	10.7	7.2	1.974081	6.1	1.808289	1.6	1	0.470004
1.526056	9.8	4.3	1.458615	7.8	2.054124	3.5	1	1.252763
1.481605	6.5	3.9	1.360977	7	1.94591	1.3	1	0.262364
1.360977	11.7	7.2	1.974081	6.2	1.824549	15.5	1	2.74084
1.526056	9.2	7.3	1.987874	5.8	1.757858	0.4	1	-0.91629
1.335001	11.7	6.9	1.931521	5.9	1.774952	2.9	1	1.064711
1.526056	9.7	5.7	1.740466	5.2	1.648659	0.4	1	-0.91629
1.547563	4.8	1.6	0.470004	4	1.386294	3.3	1	1.193922
1.458615	5.7	6.1	1.808289	5.6	1.722767	2.8	1	1.029619
1.609438	7.3	2.8	1.029619	4.4	1.481605	0.5	1	-0.69315
1.504077	10	6.3	1.84055	5.1	1.629241	0.9	1	-0.10536
1.458615	3.8	7.6	2.028148	4.2	1.435085	7.4	1	2.00148
1.308333	9.3	6.4	1.856298	10	2.302585	79.8	1	4.379524
1.458615	5.7	4.9	1.589235	4.6	1.526056	8.3	1	2.116256
1.481605	8.5	4.7	1.547563	5.8	1.757858	2.3	1	0.832909
0.955511	5.2	5.5	1.704748	4.8	1.568616	0.2	0	-1.60944
1.386294	3.7	3.7	1.308333	4.2	1.435085	30.1	1	3.404525
1.360977	16	6.7	1.902108	8.8	2.174752	7	1	1.94591
1.386294	9.4	6.4	1.856298	4.2	1.435085	3.9	1	1.360977
1.481605	10.3	6.5	1.871802	4.5	1.504077	2.3	1	0.832909
1.458615	6.8	5.4	1.686399	5.1	1.629241	6.5	1	1.871802
1.335001	8	4.4	1.481605	2.7	0.993252	14.2	1	2.653242
1.526056	8.2	6.2	1.824549	4.4	1.481605	22.1	1	3.095578
1.435085	2.7	7	1.94591	4.3	1.458615	1	1	0
1.458615	8.8	3.9	1.360977	3	1.098612	1	1	0
1.435085	6.6	3.5	1.252763	7.9	2.066863	1.2	1	0.182322
1.410987	9.8	4.2	1.435085	5.5	1.704748	3	1	1.098612
1.435085	9.3	9.3	2.230014	8.6	2.151762	2.3	1	0.832909

1.386294	4.7	8	2.079442	4.4	1.481605	47.6		1	3.862833
1.410987	15.6	9.7	2.272126	4.1	1.410987	12.3		1	2.509599
1.547563	11.9	7.2	1.974081	11.9	2.476538	1.6		1	0.470004
1.547563	12.4	9.4	2.24071	8.1	2.091864	2.6		1	0.955511
1.481605	5.4	5.4	1.686399	3.9	1.360977	0.1		0	-2.30259
1.360977	3.7	8.4	2.128232	6.1	1.808289	1.3		1	0.262364
1.481605	7.7	4.5	1.504077	4.6	1.526056	3.1		1	1.131402
1.360977	7.6	3.6	1.280934	4.4	1.481605	6.6		1	1.88707
1.435085	4.2	7	1.94591	2.8	1.029619	2		1	0.693147
1.131402	8	5.9	1.774952	5.1	1.629241	0.6		1	-0.51083
1.435085	8.8	4.3	1.458615	5.2	1.648659	4.2		1	1.435085
1.410987	4.5	9.7	2.272126	6.5	1.871802	1.7		1	0.530628
1.526056	9.6	4.2	1.435085	5.9	1.774952	5.2		1	1.648659
1.360977	11.9	7.7	2.04122	5.2	1.648659	1.2		1	0.182322
1.435085	4.7	7.5	2.014903	3.3	1.193922				
1.481605	3.6			3.8	1.335001				
1.386294	3.8	4	1.386294	5	1.609438	5.5		1	1.704748
1.360977	5.1	13.2	2.580217	3.8	1.335001	12.4		1	2.517696
1.360977	4.4	8.9	2.186051	5	1.609438	67.2		1	4.207673
1.547563	12.3	7.2	1.974081	6.6	1.88707	14.1		1	2.646175
1.526056	7.1	6.3	1.84055	5.5	1.704748	12.2		1	2.501436
1.504077	16.1	8.4	2.128232	8.8	2.174752	0.9		1	-0.10536
1.335001	4	13.5	2.60269	6.1	1.808289	2.9		1	1.064711
1.458615	2.8	8.8	2.174752	4.1	1.410987	1.1		1	0.09531
1.386294	3.1	14.1	2.646175	4.1	1.410987	26.9		1	3.292126
	4	8.3	2.116256	5.2	1.648659	8.2		1	2.104134
1.410987	2.6	9.3	2.230014	3.9	1.360977	2.7		1	0.993252
1.568616	6.9	6	1.791759	4.8	1.568616	2.4		1	0.875469
1.458615	4.2	8.2	2.104134	3.8	1.335001	1.1		1	0.09531
1.435085	6.4	4.6	1.526056	5.3	1.667707	0.3		0	-1.20397
1.386294	2	5.6	1.722767	3.6	1.280934	6		1	1.791759
1.547563	9.7	5.4	1.686399	4.5	1.504077	0.4		1	-0.91629
1.589235	5.6	7.4	2.00148	6	1.791759				
1.481605	5.7	4.7	1.547563	4.3	1.458615	0.6		1	-0.51083
1.504077	11	4.4	1.481605	7.9	2.066863	2.6		1	0.955511
1.335001	2.7	6.5	1.871802	3.7	1.308333	0.7		1	-0.35667
1.435085	8.4	12.5	2.525729	5.5	1.704748	13.9		1	2.631889
1.410987	5.6	7.9	2.066863	4.6	1.526056	0.6		1	-0.51083
1.386294	7.2			5.6	1.722767	5.2		1	1.648659
1.308333	3.6	5.8	1.757858	4.5	1.504077	3.7		1	1.308333
1.163151	4	6.8	1.916923	6.3	1.84055	0.7		1	-0.35667
1.504077	3	7	1.94591	3.9	1.360977	0.7		1	-0.35667
1.435085	7	3.7	1.308333	6.4	1.856298	10.2		1	2.322388
1.252763	5.8	3.1	1.131402	4.2	1.435085	1.4		1	0.336472
1.131402	10.9	4.9	1.589235	3.5	1.252763	23.5		1	3.157
1.410987	4.4	8.2	2.104134	5.6	1.722767				
1.335001	3.4	10.5	2.351375	3.5	1.252763	162.7		1	5.091908

1.458615	4.2	3.4	1.223775	3.3	1.193922	0.8	1	-0.22314
1.547563	4.1	5.4	1.686399	4.3	1.458615	0.8	1	-0.22314
1.481605	1.6	13.3	2.587764	4.6	1.526056	11.4	1	2.433613
1.252763	3.7	9.2	2.219203	3.8	1.335001	13.7	1	2.617396
1.458615	3.5	7.9	2.066863	3.7	1.308333	0.4	1	-0.91629
1.504077	2.9	8.5	2.140066	3.4	1.223775	6.4	1	1.856298
1.280934	4.1	9.6	2.261763	5.1	1.629241	5.9	1	1.774952
1.589235	6.9	6.5	1.871802	6.6	1.88707	0.7	1	-0.35667
1.386294	4	6	1.791759	4.5	1.504077	1.6	1	0.470004
1.308333	4.7	8.5	2.140066	4.3	1.458615	4.6	1	1.526056
1.308333	7.8	6.2	1.824549	4.3	1.458615	1.1	1	0.09531
1.308333	2.8	6.8	1.916923	3.5	1.252763	1.1	1	0.09531
1.589235	4.5	8.9	2.186051	4.6	1.526056	0.9	1	-0.10536
1.458615	3.7	6.8	1.916923	3	1.098612	2.3	1	0.832909
1.435085	2.4	8.8	2.174752	4.4	1.481605	2	1	0.693147
1.458615	8.3	5.1	1.629241	6.1	1.808289	8.3	1	2.116256
1.223775	10.9	7.3	1.987874	4.8	1.568616	69.1	1	4.235555
1.410987	6.7	4.9	1.589235	4.3	1.458615	3.4	1	1.223775
1.335001	4.2	9.7	2.272126	5.9	1.774952	4.1	1	1.410987
1.481605	3.5	4.8	1.568616	5	1.609438	0.4	1	-0.91629
1.481605	5.5	11.6	2.451005	4.3	1.458615	6	1	1.791759
1.547563	9.2	4.8	1.568616	2.5	0.916291	1.6	1	0.470004
1.410987	3.3	7.9	2.066863	4.8	1.568616	0.6	1	-0.51083
1.410987	2.9	9	2.197225	4	1.386294	5.6	1	1.722767
1.335001	5.3	7.7	2.04122	5.5	1.704748	1.3	1	0.262364
1.435085	2.5	9.5	2.251292	4.1	1.410987	0.5	1	-0.69315
1.360977	9.8	7.6	2.028148	3.7	1.308333	14	1	2.639057
1.589235	4.5	8.4	2.128232	4.1	1.410987	1.1	1	0.09531
1.481605	6.3	8	2.079442	4.9	1.589235	3.8	1	1.335001
1.435085	4.7	8.3	2.116256	5.6	1.722767	3.3	1	1.193922
1.360977	4.9	4.6	1.526056	4.1	1.410987	2.1	1	0.741937
1.308333	10.8	6.4	1.856298	6.9	1.931521	8.7	1	2.163323
1.435085	4.1	9.1	2.208274	4.3	1.458615	0.2	0	-1.60944
1.386294	4.2	5.8	1.757858	4.3	1.458615	2.1	1	0.741937
1.223775	10.4	5.2	1.648659	4.1	1.410987			
1.504077	6.9	6.6	1.88707	5.1	1.629241	4.2	1	1.435085
1.589235	5	3.5	1.252763	4.1	1.410987	2.1	1	0.741937
1.458615	3.9	6.7	1.902108	5.7	1.740466	2.3	1	0.832909
1.098612	4.3	7.2	1.974081	5	1.609438	0.4	1	-0.91629
1.458615	15.3	6.2	1.824549	6.4	1.856298			
1.410987	2.8	6.4	1.856298	4.9	1.589235	2.4	1	0.875469
1.547563	5.7	4.5	1.504077	5.6	1.722767	0.7	1	-0.35667
1.252763	6.3	4	1.386294	5.8	1.757858	2.1	1	0.741937
1.410987	5.8	5.9	1.774952	4.8	1.568616	0.4	1	-0.91629
1.410987	2.9	8.7	2.163323	5.1	1.629241	2.2	1	0.788457
1.410987	5.7	7.3	1.987874	5	1.609438	7.2	1	1.974081
1.481605	13.1	7.2	1.974081	13.4	2.595255	1.6	1	0.470004

1.386294	2.9	8.2	2.104134	3.4	1.223775	15.7	1	2.753661
1.386294	2.6	6.7	1.902108	3.9	1.360977	4.7	1	1.547563
1.223775	5.5	5.8	1.757858	4.3	1.458615	0.7	1	-0.35667
1.386294	3.9	6.9	1.931521	5.1	1.629241	2.3	1	0.832909
1.458615	2.7	4.8	1.568616	3.9	1.360977	23	1	3.135494
1.098612	4.3	6.3	1.84055	5.2	1.648659	0.1	0	-2.30259
1.308333	3.3	5.5	1.704748	4.6	1.526056	2.1	1	0.741937
1.410987	2.5	6.5	1.871802	3.4	1.223775	15.6	1	2.747271
1.223775	4.7	6.3	1.84055	3.6	1.280934	18.3	1	2.906901
1.223775	10.1	6.8	1.916923	4.3	1.458615	24.4	1	3.194583
1.458615	3.9	6.6	1.88707	4.3	1.458615	8.9	1	2.186051
1.280934	2.5	8.4	2.128232	4.3	1.458615	11.7	1	2.459589
1.547563	7.8	3.9	1.360977	5.6	1.722767	0.5	1	-0.69315
1.410987	9.4	5	1.609438	4.7	1.547563	0.5	1	-0.69315
1.481605	3	8.3	2.116256	3.7	1.308333	0.5	1	-0.69315
1.280934	11	5.7	1.740466	4.6	1.526056	2.9	1	1.064711
1.435085	4	8.7	2.163323	5.8	1.757858	1.9	1	0.641854
1.360977	9.1	6.8	1.916923	5.8	1.757858			
1.410987	6.8	8.4	2.128232	6	1.791759	1.2	1	0.182322
1.308333	4	7.9	2.066863	4.8	1.568616	0.7	1	-0.35667
1.410987	4.7	9.4	2.24071	4.9	1.589235	0.2	0	-1.60944
1.458615	7.7	4	1.386294	6.2	1.824549	7.2	1	1.974081
1.335001	4.2	9.2	2.219203	4.2	1.435085	1.3	1	0.262364
1.435085	6.2	3.7	1.308333	4.5	1.504077			
1.435085	6	8.8	2.174752	4.1	1.410987	0.3	0	-1.20397
1.360977	4.1	6.6	1.88707	3.7	1.308333	2.6	1	0.955511
1.335001	3.8	7.7	2.04122	5.3	1.667707	19.4	1	2.965273
1.131402	3.8	4.4	1.481605	3	1.098612	30.4	1	3.414443
1.609438	7	5.3	1.667707	4.8	1.568616	2	1	0.693147
1.223775	6.8	6.3	1.84055	7.5	2.014903	4.4	1	1.481605
1.458615	6.1	6.1	1.808289	4.7	1.547563	34.4	1	3.538057
1.481605	11.2	6.7	1.902108	5.3	1.667707	0.6	1	-0.51083
1.252763	2.8	6.3	1.84055	3.3	1.193922	6.8	1	1.916923
1.252763	10.3	5.2	1.648659	5.1	1.629241	5.14	1	1.637053
1.335001	4	3.8	1.335001	5.3	1.667707	1.1	1	0.09531
1.481605	3.1	6.6	1.88707	3.3	1.193922			
1.410987	7	5.7	1.740466	3.6	1.280934	11.9	1	2.476538
1.386294	5	9.7	2.272126	5.9	1.774952	1.6	1	0.470004
1.280934	4.2	8.4	2.128232	4.8	1.568616	2.4	1	0.875469
1.252763	2.4	7.9	2.066863	3.5	1.252763	2.9	1	1.064711
1.360977	5.2	5.1	1.629241	3.6	1.280934	38.4	1	3.648057
1.223775	9.4	9.4	2.24071	5.2	1.648659	39	1	3.663562
1.064711	2.2	8.7	2.163323	3.2	1.163151	7.4	1	2.00148
1.458615	5.2	5.4	1.686399	4.5	1.504077	19.5	1	2.970414
1.526056	10.4	5.5	1.704748	3.6	1.280934	1.2	1	0.182322
1.435085	8	6.5	1.871802	5.6	1.722767	17.5	1	2.862201
1.589235	11.8	9	2.197225	4	1.386294	4	1	1.386294

1.458615	5.1	9.4	2.24071	5.5	1.704748	1.3	1	0.262364
1.280934	11.8	6.2	1.824549	6.4	1.856298	0.4	1	-0.91629
1.435085	4.7	5.8	1.757858	4.4	1.481605	2.8	1	1.029619
1.458615	4.5	4	1.386294	4.6	1.526056	0.7	1	-0.35667
1.629241	5.6	5.1	1.629241	6.1	1.808289	2	1	0.693147
1.547563	7.8	3.9	1.360977	6.2	1.824549	19	1	2.944439
1.481605	8	4.4	1.481605	5.6	1.722767	0.8	1	-0.22314
1.458615	5.5	5.2	1.648659	4.4	1.481605	3.6	1	1.280934
1.360977	8.3	5.9	1.774952	6	1.791759	0.2	0	-1.60944
1.410987	3.9	9.7	2.272126	5	1.609438	34.7	1	3.54674
1.386294	14.6	5.7	1.740466	6.1	1.808289	14.2	1	2.653242
1.335001	6	9.4	2.24071	4.8	1.568616	1.6	1	0.470004
1.410987	10.5	3.9	1.360977	6.3	1.84055	9.8	1	2.282382

cTnT	cTnT >0.01	log cTnT	sST2	sST2>=30	log sST2	PTH	logPTH	CAD
0.005	0	-5.29832	28.2	0	3.339322	550	6.309918	n
0.04	1	-3.21888	25	0	3.218876	120	4.787492	n
0.05	1	-2.99573	38.6	1	3.653252	513	6.240276	n
0.03	1	-3.50656	42.4	1	3.747148	888	6.788972	n
0.03	1	-3.50656	33.8	1	3.520461	160	5.075174	n
0.005	0	-5.29832	11.8	0	2.4681	66	4.189655	n
0.08	1	-2.52573	45.4	1	3.815512	308	5.7301	y
0.005	0	-5.29832	13.7	0	2.617396	47	3.850148	n
0.005	0	-5.29832	15.8	0	2.76001	345	5.843544	n
0.18	1	-1.7148	32.2	1	3.471966	95	4.553877	n
0.02	1	-3.91202	31.8	1	3.459466	250	5.521461	n
0.02	1	-3.91202	34.4	1	3.538057	219	5.389072	n
0.005	0	-5.29832	27	0	3.295837	145	4.976734	n
0.005	0	-5.29832	46.7	1	3.843744	169	5.129899	n
0.17	1	-1.77196	34	1	3.526361	194	5.267858	y
0.005	0	-5.29832	13.6	0	2.61007	97	4.574711	n
0.02	1	-3.91202	14.7	0	2.687847	242	5.488938	n
0.03	1	-3.50656	26.1	0	3.261935	565	6.336826	y
0.04	1	-3.21888	31.2	1	3.440418	165	5.105945	y
0.01	1	-4.60517	25.9	0	3.254243	982	6.889591	n
0.005	0	-5.29832	17.5	0	2.862201	77	4.343805	n
0.1	1	-2.30259	47.9	1	3.869116	540	6.291569	y
0.005	0	-5.29832	14.6	0	2.681022	467	6.146329	n
0.05	1	-2.99573	22	0	3.091042	230	5.438079	n
0.02	1	-3.91202	29.7	0	3.391147	349	5.855072	n
0.03	1	-3.50656	45.7	1	3.822098	264	5.575949	n
0.1	1	-2.30259	33.1	1	3.499533	548	6.306275	n
0.005	0	-5.29832	19.3	0	2.960105	232	5.446737	n
0.11	1	-2.20727	29.6	0	3.387774	149	5.003946	n
0.005	0	-5.29832	17.4	0	2.85647	56	4.025352	n
0.01	1	-4.60517	16	0	2.772589	225	5.4161	n
0.05	1	-2.99573	42.9	1	3.758872	274	5.613128	y
0.04	1	-3.21888	16	0	2.772589	92	4.521789	n
0.005	0	-5.29832	23.6	0	3.161247	29	3.367296	n
0.005	0	-5.29832	36.6	1	3.600048	348	5.852202	n
0.02	1	-3.91202	32	1	3.465736	499	6.212606	n
0.04	1	-3.21888	16.5	0	2.80336	234	5.455321	n
0.02	1	-3.91202	43.9	1	3.781914	388	5.961005	n
0.005	0	-5.29832	18	0	2.890372	208	5.337538	y
0.07	1	-2.65926	18.7	0	2.928524	436	6.077642	n
0.28	1	-1.27297	50.3	1	3.918005	172	5.147494	y
0.005	0	-5.29832	12.6	0	2.533697	96	4.564348	n
0.23	1	-1.46968	14.9	0	2.701361	340	5.828946	n
0.005	0	-5.29832	15.8	0	2.76001	14	2.639057	n
0.02	1	-3.91202	31	1	3.433987	133	4.890349	n
0.02	1	-3.91202	24.8	0	3.210844	511	6.23637	n

0.005	0	-5.29832	29.2	0	3.374169	201	5.303305	n
0.005	0	-5.29832	5.9	0	1.774952	209	5.342334	n
0.03	1	-3.50656	47.2	1	3.854394	229	5.433722	n
0.04	1	-3.21888	34.7	1	3.54674	487	6.188264	n
0.03	1	-3.50656	29	0	3.367296	119	4.779123	n
0.005	0	-5.29832	29.1	0	3.370738	305	5.720312	n
0.19	1	-1.66073	33.3	1	3.505557	541	6.293419	y
0.005	0	-5.29832	17.8	0	2.879198	454	6.118097	n
0.02	1	-3.91202	44.8	1	3.802208	486	6.186209	n
0.19	1	-1.66073	27.7	0	3.321432	86	4.454347	n
0.03	1	-3.50656	26.2	0	3.265759	355	5.872118	n
0.03	1	-3.50656	365	1	5.899897	856	6.75227	y
0.15	1	-1.89712	50.1	1	3.914021	240	5.480639	y
0.04	1	-3.21888	40.8	1	3.708682	487	6.188264	n
0.06	1	-2.81341	33.8	1	3.520461	108	4.682131	y
0.005	0	-5.29832	13.6	0	2.61007	80	4.382027	y
0.03	1	-3.50656	6.8	0	1.916923	23	3.135494	y
0.005	0	-5.29832	21.6	0	3.072693	240	5.480639	n
0.08	1	-2.52573	75.7	1	4.326778	329	5.796058	n
0.01	1	-4.60517	46.4	1	3.837299	499	6.212606	n
0.07	1	-2.65926	56.3	1	4.030695	74	4.304065	n
0.01	1	-4.60517	14	0	2.639057	864	6.761573	y
0.03	1	-3.50656	55.3	1	4.012773	536	6.284134	y
0.005	0	-5.29832	20.3	0	3.010621	127	4.844187	n
0.005	0	-5.29832	62.7	1	4.138361	228	5.429346	n
0.08	1	-2.52573	18.4	0	2.912351	323	5.777652	n
0.06	1	-2.81341	34	1	3.526361	16	2.772589	y
0.26	1	-1.34707	20.5	0	3.020425	268	5.590987	y
0.005	0	-5.29832	5.1	0	1.629241	80	4.382027	n
0.005	0	-5.29832	6.3	0	1.84055	111	4.70953	n
0.005	0	-5.29832	15.2	0	2.721295	120	4.787492	n
0.005	0	-5.29832	34.6	1	3.543854	626	6.43935	n
0.005	0	-5.29832	48.1	1	3.873282	367	5.905362	n
0.005	0	-5.29832	16.5	0	2.80336	188	5.236442	n
0.02	1	-3.91202	20.4	0	3.015535	382	5.945421	n
0.005	0	-5.29832	10.4	0	2.341806	86	4.454347	n
0.78	1	-0.24846	43.4	1	3.770459	149	5.003946	n
0.005	0	-5.29832	33.1	1	3.499533	572	6.349139	y
0.005	0	-5.29832	19.3	0	2.960105	307	5.726848	n
0.04	1	-3.21888	40.1	1	3.691376	33	3.496508	n
0.005	0	-5.29832	16.1	0	2.778819	380	5.940171	n
0.01	1	-4.60517	30.5	1	3.417727	18	2.890372	y
0.26	1	-1.34707	55.9	1	4.023564	1355	7.211557	n
0.005	0	-5.29832	15.3	0	2.727853	245	5.501258	n
0.19	1	-1.66073	40.7	1	3.706228	36	3.583519	y
0.1	1	-2.30259	33.9	1	3.523415	207	5.332719	y
0.32	1	-1.13943	152.5	1	5.027165	35	3.555348	y

0.02	1	-3.91202	19.3	0	2.960105	490	6.194405	n
0.01	1	-4.60517	13.4	0	2.595255	353	5.866468	n
0.005	0	-5.29832	16.5	0	2.80336	77	4.343805	y
0.005	0	-5.29832	19.3	0	2.960105	367	5.905362	n
0.005	0	-5.29832	28	0	3.332205	121	4.795791	n
0.005	0	-5.29832	25.4	0	3.234749	62	4.127134	n
0.07	1	-2.65926	51	1	3.931826	414	6.025866	n
0.005	0	-5.29832	37.8	1	3.632309	202	5.308268	n
0.005	0	-5.29832	36.5	1	3.597312	27	3.295837	n
0.005	0	-5.29832	44.2	1	3.788725	183	5.209486	n
0.02	1	-3.91202	22.2	0	3.100092	42	3.73767	n
0.005	0	-5.29832	22.2	0	3.100092	198	5.288267	n
0.03	1	-3.50656	27.7	0	3.321432	376	5.929589	n
0.005	0	-5.29832	23.6	0	3.161247	74	4.304065	n
0.005	0	-5.29832	22.1	0	3.095578	115	4.744932	n
0.14	1	-1.96611	27.8	0	3.325036	200	5.298317	n
0.03	1	-3.50656	31.5	1	3.449988	213	5.361292	n
0.07	1	-2.65926	29.1	0	3.370738	147	4.990433	y
0.2	1	-1.60944	46.2	1	3.83298	27	3.295837	n
0.005	0	-5.29832	21.6	0	3.072693	254	5.537334	n
0.04	1	-3.21888	37.6	1	3.627004	89	4.488636	n
0.02	1	-3.91202	30.3	1	3.411148	209	5.342334	n
0.005	0	-5.29832	28.4	0	3.346389	82	4.406719	n
0.005	0	-5.29832	21.9	0	3.086487	33	3.496508	n
0.005	0	-5.29832	33.4	1	3.508556	304	5.717028	n
0.02	1	-3.91202	24.3	0	3.190476	66	4.189655	n
0.02	1	-3.91202	22.7	0	3.122365	159	5.068904	n
0.005	0	-5.29832	36.5	1	3.597312	95	4.553877	n
0.06	1	-2.81341	42.3	1	3.744787	294	5.68358	y
0.005	0	-5.29832	39.8	1	3.683867	157	5.056246	n
0.005	0	-5.29832	55.4	1	4.01458	100	4.60517	n
0.03	1	-3.50656	26.3	0	3.269569	1348	7.206377	n
0.005	0	-5.29832	23.8	0	3.169686			n
0.005	0	-5.29832	29.9	0	3.397858	180	5.192957	n
0.005	0	-5.29832	15.7	0	2.753661	270	5.598422	n
0.005	0	-5.29832	33.8	1	3.520461	157	5.056246	n
0.03	1	-3.50656	39.3	1	3.671225	103	4.634729	y
0.08	1	-2.52573	29.9	0	3.397858	177	5.17615	n
0.08	1	-2.52573	26.4	0	3.273364	366	5.902633	n
0.07	1	-2.65926	161.1	1	5.082025	255	5.541264	n
0.02	1	-3.91202	26.5	0	3.277145	14	2.639057	n
0.005	0	-5.29832	18.4	0	2.912351	85	4.442651	n
0.005	0	-5.29832	21.3	0	3.058707	218	5.384495	n
0.02	1	-3.91202	41.4	1	3.723281	155	5.043425	y
0.03	1	-3.50656	26.1	0	3.261935	15	2.70805	n
0.005	0	-5.29832	14.2	0	2.653242	160	5.075174	n
0.08	1	-2.52573	69	1	4.234107	574	6.352629	n

0.005	0	-5.29832	15.9	0	2.766319	275	5.616771	n
0.005	0	-5.29832	27.4	0	3.310543	93	4.532599	y
0.02	1	-3.91202	37.8	1	3.632309	140	4.941642	y
0.07	1	-2.65926	31.6	1	3.453157	201	5.303305	y
0.005	0	-5.29832	25.9	0	3.254243	55	4.007333	n
0.005	0	-5.29832	43.9	1	3.781914	138	4.927254	n
0.005	0	-5.29832	24.1	0	3.182212	159	5.068904	n
0.005	0	-5.29832	18.4	0	2.912351	153	5.030438	n
0.02	1	-3.91202	20.65	0	3.027715	204	5.31812	n
0.1	1	-2.30259	89.5	1	4.494239	191	5.252273	n
0.06	1	-2.81341	18.7	0	2.928524	47	3.850148	n
0.005	0	-5.29832	48.8	1	3.88773	30	3.401197	n
0.02	1	-3.91202	24.6	0	3.202746	367	5.905362	n
0.01	1	-4.60517	25.8	0	3.250374	518	6.249975	n
0.005	0	-5.29832	13.3	0	2.587764	115	4.744932	n
0.3	1	-1.20397	32.9	1	3.493473	236	5.463832	n
0.005	0	-5.29832	23.2	0	3.144152	243	5.493061	n
0.17	1	-1.77196	17.3	0	2.850707	401	5.993961	y
0.005	0	-5.29832	34.1	1	3.529297	184	5.214936	n
0.005	0	-5.29832	26.2	0	3.265759	98	4.584967	n
0.22	1	-1.51413	13.6	0	2.61007	446	6.100319	n
0.005	0	-5.29832	48.8	1	3.88773	180	5.192957	n
0.03	1	-3.50656	29.1	0	3.370738	288	5.66296	y
0.04	1	-3.21888	22.9	0	3.131137	743	6.610696	n
0.15	1	-1.89712	37.3	1	3.618993	139	4.934474	n
0.005	0	-5.29832	27.8	0	3.325036	57	4.043051	y
0.005	0	-5.29832	53.3	1	3.975936	151	5.01728	n
0.12	1	-2.12026	154.4	1	5.039547	44	3.78419	n
0.01	1	-4.60517	14.3	0	2.66026	405	6.003887	n
0.19	1	-1.66073	25.1	0	3.222868	237	5.46806	y
0.05	1	-2.99573	21.4	0	3.063391	170	5.135798	n
0.32	1	-1.13943	36.8	1	3.605498	299	5.700444	n
0.07	1	-2.65926	168.6	1	5.127529			y
0.005	0	-5.29832	18.7	0	2.928524	462	6.135565	n
0.05	1	-2.99573	20.2	0	3.005683	166	5.111988	y
0.005	0	-5.29832	9.7	0	2.272126	172	5.147494	n
0.005	0	-5.29832	28.4	0	3.346389	109	4.691348	n
0.16	1	-1.83258	32.3	1	3.475067	290	5.669881	n
0.03	1	-3.50656	13.3	0	2.587764	166	5.111988	n
0.005	0	-5.29832	36.4	1	3.594569	73	4.290459	n
0.05	1	-2.99573	18.7	0	2.928524	193	5.26269	n
0.12	1	-2.12026	350	1	5.857933	327	5.78996	n
0.005	0	-5.29832	49.6	1	3.903991	116	4.75359	n
0.005	0	-5.29832	10.7	0	2.370244	242	5.488938	n
0.03	1	-3.50656	21.2	0	3.054001	87	4.465908	y
0.18	1	-1.7148	93.7	1	4.540098	29	3.367296	n
0.005	0	-5.29832	14.9	0	2.701361	210	5.347108	n

0.005	0	-5.29832	25.5	0	3.238678	236	5.463832	n
0.1	1	-2.30259	22.1	0	3.095578	545	6.300786	n
0.005	0	-5.29832	22.7	0	3.122365	548	6.306275	n
0.005	0	-5.29832	19	0	2.944439	369	5.910797	n
0.005	0	-5.29832	32.2	1	3.471966	415	6.028279	n
0.44	1	-0.82098	23.5	0	3.157	588	6.376727	y
0.01	1	-4.60517	23.3	0	3.148453	343	5.83773	y
0.01	1	-4.60517	31.5	1	3.449988	150	5.010635	n
0.05	1	-2.99573	46.7	1	3.843744	42	3.73767	n
0.005	0	-5.29832	50	1	3.912023	38	3.637586	n
0.005	0	-5.29832	27.9	0	3.328627	416	6.030685	y
0.14	1	-1.96611	102.5	1	4.629863	371	5.916202	n
0.005	0	-5.29832	32.6	1	3.484312	114	4.736198	n

AMI_Pre	CHF_Pre	Stroke_Pre	Carotid_Pr	RenalAS_P	PVD_Pre	HxCAD/CH	DobutEcho	LVEF
n		n	n	n	n	0	0	65
n	n	n	n	n	n	0	0	60
n	n	n	n	y	n	0	1	52
n	n	n	n	n	n	0	0	67
n	y	n	n	n	y	1		51
n		n	n	n	n	0		
y	n	n	n	n	n	1	0	75
n	n	n	n	n	n	0	0	57
n	n	n	n	n	n	0	0	58
n	n	n	n	n	n	0		69
n		n	n	n	n	0	0	60
n	n	y	n	n	y	1	0	66
n	n	n	n	n	n	0	0	66
n		n	n	n	n	0	0	65
y	n	n	n	n	n	1	0	59
n		n	n	n	n	0	0	65
n	n	n	n	n	n	0	0	60
n	n	n	n	n	y	1	0	65
n	n	n	n	n	n	1	1	53
n	n	n	n	n	n	0	0	65
n	n	n	n	n	n	0	0	64
n	n	n	n	n	n	1	1	60
n	n	n	n	n	n	0	0	68
n	n	n	n	n	n	0	0	65
n	n	n	n	n	n	0	0	59
n	n	n	n	n	n	0	0	64
n	n	n	n	n	y	1	0	60
n	n	n	n	n	n	0	0	59
n	n	n	n	n	n	0	1	45
n	n	n	n	n	n	0	0	60
n	n	n	n	n	n	0	0	65
y	n	n	n	n	n	1		34
n	y	n	n	n	n	1	0	65
n	n	n	n	n	n	0	0	62
n	y	n	n	n	n	1	0	60
n	n	n	n	n	n	0	0	
n	n	n	n	n	n	0	0	68
n	n	n	n	n	n	0	1	55
y	n	n	n	n	n	1	0	47
n	n	y	n	n	n	1	0	64
n	n	n	n	n	n	1	1	40
n	n	n	n	n	n	0	0	62
n	n	n	n	n	n	0	0	67
n	n	n	n	n	y	1	0	55
n	n	n	n	n	n	1	1	61
n	n	n	n	n	n	0	0	60

n	n	n	n	n	y	1	0	65
n	n	n	n	n	n	0		65
n	y	n	n	n	n	1		65
n	n	n	n	n	n	0	0	65
n	n	n	n	n	y	1	0	66
n		n	n	n	n	0	0	64
n	n	n	n	n	n	1	0	59
n	n	n	n	n	n	0	0	66
n	n	n	n	n	n	0	0	65
n	y	n	n	n	y	1	0	78
n	n	n	n	n	n	0	0	64
y	n	n	n	n	n	1		63
n	n	n	n	n	n	1	0	63
n	n	n	n	n	n	0	0	56
n	n	n	n	n	n	1		
n	y	n	n	n	y	1	1	67
n	n	n	y	n	y	1	0	66
n	n	n	n	n	n	0	0	66
n	n	n	n	n	n	0	0	65
n	y	n	n	n	n	1	1	32
n	n	n	n	n	y	1	0	65
n	n	n	n	n	n	1		53
y	y	y	n	n	n	1	0	63
n	n	n	n	n	n	0	0	67
n	n	n	n	n	n	0		
n	n	n	n	n	n	0		75
n	y	n	n	n	n	1		30
n	y	n	n	n	n	1	1	65
n	n	n	n	n	n	0		
n	n	n	n	n	n	0		
n	n	n	n	n	n	0	0	59
n	n	n	n	n	n	0	0	60
n	n	n	n	n	n	0		
n	n	n	n	n	n	0	0	66
n	n	n	n	n	n	0	0	56
n	n	n	n	n	n	0		
n	n	n	n	n	n	0	0	65
n	n	n	n	n	n	1	0	65
n	n	n	n	n	n	0		
n	n	n	n	n	n	0	0	61
n	n	n	n	n	n	0		
y	n	n	n	n	n	1	0	60
n	n	n	n	n	n	0	1	62
n	n	n	n	n	y	1	1	66
n	y	n	n	n	n	1	1	45
n	y	n	n	n	n	1		37
n	n	n	n	n	n	0	0	63

n	n	n	n	n	n	0	0	65
n	n	n	n	n	n	0	0	67
n	y	n	n	n	n	1		22
n	n	n	n	n	n	0		
n	n	n	n	n	n	0	0	65
n	n	n	n	n	n	0	0	65
n	y	n	n	y	y	1	1	45
n	n	n	n	n	n	0	0	65
n	n	n	n	n	n	0	0	65
n	n	n	n	n	n	0	0	60
n	n	n	n	n	n	0	0	55
n	n	n	n	n	n	0		
n	n	n	n	n	n	0		69
n	n	n	n	n	n	0	0	58
n	n	n	n	n	n	0	0	65
n	n	n	n	n	n	0	0	53
n	n	n	n	n	n	0		63
n	y	y	n	n	n	1	1	60
n	y	y	n	n	n	1	0	60
n	n	n	n	n	n	0	0	65
n	n	n	n	n	n	0		
n	n	y	n	n	n	1	0	65
n	n	n	n	n	n	0	0	42
n	n	n	n	n	n	0	1	63
n	n	n	n	n	n	0		65
n	n	n	n	n	n	0	0	65
n	n	n	n	n	n	0	0	55
n	n	n	n	n	n	0	0	54
y	y	n	y	n	n	1	0	49
n	n	y	n	n	n	1	0	54
n	n	n	n	n	y	1	0	64
n	n	n	n	n	n	0		62
n	n	n	n	n	n	0		
n	n	n	n	n	n	0	0	65
n	n	n	n	n	n	0		61
n	n	n	n	n	n	0	0	63
y	n	n	n	n	n	1	0	65
n	n	n	n	n	n	0	0	42
n	n	n	n	n	n	0	0	66
n	n	n	n	n	n	0	1	40
n	n	n	n	y	n	0	0	65
n	n	n	n	n	n	0		
n	n	n	n	n	n	0	0	64
n	n	n	n	n	y	1	0	65
n	y	y	n	n	n	1	0	60
n	n	n	n	n	n	0		
n	n	n	n	n	n	0	0	57

n	n	n	n	n	n	0		
n	n	n	n	n	y	1	0	64
y	n	n	n	n	n	1	1	62
n	n	n	n	n	n	1	1	62
n	n	n	n	n	n	0	0	66
n	n	n	n	n	n	0		
n	n	n	n	n	n	0		
n	n	n	n	n	n	0	0	66
n	n	n	n	n	n	0	1	63
n	n	n	n	n	n	0	0	74
n	n	n	n	n	n	0	0	64
n	n	n	n	n	n	0		69
n	n	y	n	n	n	1	0	64
n	n	n	n	n	n	0	0	61
n	n	n	n	n	n	0	0	65
n	n	y	n	n	n	1	0	62
n	n	n	n	n	n	0	0	65
n	n	n	n	n	n	1	0	64
n	n	y	n	n	n	1	0	64
n	n	n	n	n	n	0		
n	n	n	n	n	n	0	0	65
n	n	n	n	n	n	0	0	65
y	n	n	y	n	n	1	0	60
n	n	n	n	n	n	0	0	56
n	n	n	n	n	n	0	0	65
y	n	n	n	y	n	1	0	60
n	n	n	n	n	n	0	0	67
n	n	n	n	n	n	0		61
n	n	n	n	n	n	0	0	65
y	n	n	n	n	n	1	0	66
n	n	n	n	n	n	0	0	64
n	n	n	n	n	n	0	0	65
y	n	y	n	n	n	1		65
n	n	n	n	n	n	0		59
n	n	n	n	n	n	1		49
n	n	n	n	n	n	0	0	50
n	n	n	n	n	n	0	0	65
n	n	n	y	n	y	1	1	65
n	n	n	n	n	n	0		
n	n	n	n	n	n	0	0	65
n	y	n	n	n	n	1		60
n	n	n	n	n	n	0	0	72
n	n	n	n	n	n	0	0	70
n	n	n	n	n	n	0	0	65
n	n	n	n	n	n	1	1	66
n	n	n	n	n	y	1	0	52
n	n	n	n	n	n	0		

n	n	n	n	n	n	0	0	65
n	n	n	n	n	n	0		58
n	n	n	n	n	n	0		65
n	n	n	n	n	n	0	0	65
n	n	n	n	n	n	0	0	65
y	n	n	n	n	n	1	0	65
n	n	n	n	n	n	1	1	65
n	n	n	n	n	n	0	1	57
n	n	n	n	n	n	0	0	65
n	n	n	n	n	n	0	0	65
y	n	n	n	n	n	1	0	49
n	n	n	n	n	n	0		70
n	n	n	n	n	n	0	0	70

EF<45	LVH	LVMI	LVMI>103	LVMI>89 w	LVH-LVMI r	LVM	OrganStatu	DonorType
0	1	162	1	0	1	430	Active	LRD
0	0	46	0	0	0	75	Active	CAD
0				0				
0	0	84	0	0	0	181		
0	0	102	0	0	0	256		
				0			Active	LURD
0	1	155	1	0	1	357	Active	LURD
0	0	82	0	0	0	175		LRD
0	0	82	0	0	0	109	Active	LRD
0	1	245	1	0	1	108		
0	0	100	0	0	0	219	Active	CAD
0	0	100	0	1	1	158		
0	0	105	0	0	0	220	Active	LURD
0	0	72	0	0	0	137		LRD
0	0			0				CAD
0	0	95	0	0	0	148		
0	0	140	1	0	1	248		
0	0	97	0	0	0	234		LRD
0	0	136	1	0	1	272		
0	0	86	0	0	0	170	Active	CAD
0	1	114	0	0	0	256	Active	CAD
0	0	132	1	0	1			
0	0	70	0	0	0	110	Active	LRD
0	0	69	0	0	0	137		
0	0			0				
0	0	93	0	0	0	234	Active	LURD
0	0	85	0	0	0	153		
0	0	116	0	1	1	187	Active	CAD
0	0	162	1	0	1	280		
0	0	78	0	0	0	163	Active	CAD
0	0	85	0	0	0	173		
1	0	99	0	0	0	209		LURD
0	0	109	0	1	1	175		
0	0	99	0	0	0	175	Active	LURD
0	0	98	0	0	0	163		LRD
				0				
0	0	100	0	1	1	158		LRD
0	0	116	1	0	1	227	Active	CAD
0	0	150	0	1	1	227		
0	0	89	0	0	0	169	Active	CAD
1	0	101	0	0	0	200	Active	CAD
0	0	71	0	0	0	114		
0	1	178	1	0	1	278		
0	0	99	0	0	0	194	Active	CAD
0	1	150	1	0	1	241		
0	0			0				CAD

0	0	51	0	0	0	96		
0	0			0		127	Active	LRD
0	0	141	0	1	1	219		
0	1	137	1	0	1	330		
0	1	105	0	1	1	159		LURD
0	0		0				Active	LRD
0	0	114	0	0	0	234		LURD
0	0	69	0	0	0	147		
0	1	162	1	0	1	330		
0	0	78	0	0	0	117		
0	1	100	0	0	0	226	Active	CAD
0	0	104	0	1	1	153		
0	0	95	0	0	0	200		
0	1	147	1	0	1	286	Active	CAD
				0			Active	LURD
0	0	67	0	0	0	132	Active	CAD
0	0	112	0	0	0	187	0	
0	0	101	0	0	0	249		
0	1	131	1	0	1	249	Active	CAD
1	0	145	1	0	1	288		
0	1	129	1	0	1	261		
0	0	120	1	0	1	255		
0	1	130	1	0	1	267		
0	0	80	0	0	0	123		
			0				Active	LURD
0	1	95	0	0	0	194	Active	CAD
1	1	167	1	0	1	368	Active	LURD
0	0	100	0	0	0	213	Active	LRD
				0			Active	LRD
				0			Active	LURD
0	0	65	0	0	0	109		
0	0	89	0	0	0	153		
				0			Active	
0	0	113	0	0	0	193		
0	0	123	1	0	1	211		
			0				Ac	
0	1	117	1	0	1	271		
0	0	94	0	0	0	191		
			0				Active	LRD
0	0	108	0	0	0	219		
			0				Active	LRD
0	0	61	0	0	0	106		
0	1	173	1	0	1	355		
0	0	1353	0	1	1	2922	Active	CAD
0	1	147	1	0	1	339	Active	LURD
1	0			0			Active	
0	0	107	0	0	0			

0	1	110	0	1	1	224		
0	0	113	0	0	0	219	Active	LRD
1	0	146	0	1	1	294		
			0					
0	0	101	0	0	0	213	Active	LURD
0	0	87	0	0	0	147		
0	1	158	1	0	1	334		
0	0	123	0	1	1	169	Active	LURD
0	0	87	0	0	0	181		
0	0	220	1	0	1	97		
0	0			0			Active	LRD
				0			Active	LRD
0	0	174	1	0	1	116	Active	
0	0	70	0	0	0	154	Active	LRD
0	0	107	0	1	1	234		
0				0				
0	0		0				Active	LRD
0	0	79	0	0	0	132	Active	CAD
0	1	80	0	0	0	187		
0	0	94	0	0	0	163		q
				0			Active	LURD
0	1	175	0	1	1	282	Active	CAD
1	0	135	1	0	1	268	Active	LURD
0	0	90	0	0	0	156		
0	0	95	0	0	0	175		
0	1	92	0	0	0	141		
0	1	185	1	0	1	463	Active	CAD
0	0	95	0	0	0	219	Active	LURD
0	0	67	0	0	0	127		
0	0	84	0	0	0	146	Active	LURD
0	0	91	0	0	0	158		
0	0	86	0	0	0	158	Active	CAD
				0			Active	LURD
0	0	65	0	0	0	142	Active	
0	1	120	0	1	1	194	Active	LURD
0	0	94	0	0	0	206	Active	LURD
0	0	104	0	0	0	200		
1				0			Active	
0	1	96	0	0	0	158	Active	LRD
1	0	108	0	0	0	220		
0	0		0					
			0				Active	CAD
0	0	95	0	0	0	175		
0	1	140	0	1	1	227		
0	1			0				
				0			Active	LRD
0	1	188	1	0	1	388	Active	

			0				Active	CAD
0	0		0					
0	0	106	0	1	1	194	Active	LURD
0	0	119	1	0	1	264	Active	LURD
0			0					
				0			Active	LURD
			0				Active	LURD
0	0	121	0	1	1	194		
0	1	78	0	0	0	153		
0	1	145	1	0	1	248		
0	0	91	0	0	0	193	Active	LRD
0	0	104	0	0	0	234		
0	0	99	0	0	0	173	Active	LRD
0	0	84	0	0	0	164	Active	CAD
0	0	63	0	0	0	109		
0	1	161	1	0	1	325		
0	0	74	0	0	0	132	Active	LRD
0	1	122	1	0	1	279		
0	0	80	0	0	0	158		
				0			Active	LURD
0	1	139	0	1	1	248		
0	0	64	0	0	0	123		
0	0	124	1	0	1	267		
0	0	99	0	0	0	187		
0	0	110	0	0	0	187		
0	0	92	0	0	0	181	Active	LRD
0	0	65	0	0	0	153		
0	0	86	0	0	0	181		
0	0	78	0	0	0	142	Active	CAD
0	0	88	0	0	0	164		
0	0	104	0	0	0	194	Active	LURD
0	1	148	1	0	1	276		
0	0	115	0	1	1	193	Active	LRD
0	0	83	0	0	0	145		
0	1	154	0	1	1	263		
0	0	99	0	0	0	205	Active	LURD
0	0	134	1	0	1	296	Active	CAD
0	0			0				
				0			Active	LURD
0	0	107	0	1	1	194		
0	1	127	0	1	1	245		
0	0	107	0	1	1	186	Active	CAD
0	0		0				Active	CAD
0	1	101	0	1	1	210	Active	LURD
0	0	111	0	0	0	211		
0	0	123	0	1	1	220		
				0			Active	

0	0	99	0	0	0	207	Active	LRD
0	0	96	0	0	0	181		
0	0	89	0	0	0	227		
0	0	143	0	1	1	241		
0	0	97	0	0	0	132		
0	0	112	0	0	0	272		
0	0	120	1	0	1	249		
0	0			0				
0	0	120	0	1	1	194	Active	CAD
0	1	222	1	0	1	416		
0	0	109	0	0	0	256		
0	1	161	1	0	1	352		
0	1	122	1	0	1	235		

Approval St	TxElsewher	Tx status u	transplante	CV events	CAD (0=nor	angiogram	Outcome c	CVE post-ev
1	n	1	1	0	0	0		0
1	n	1	1	0	0	0		0
1	n	2	0	0	0	0	h/o of seve	1
1	n	3	0	0	0	0	death	0
0	n	4	0	1	0	0	CHF	0
1	n	1	1	0	0	0		0
1	n	1	1	1	3	1	h/o of CAD	0
1	n	2	0	0	0	0		1
1	n	1	1	0	0	0		0
0	n	4	0	0	0	0	h/o of AF, c	0
1	y	1	1	0	0	0		0
0	n	4	0	1	0	0	CVA	1
1	n	1	1	0	0	0		0
1	n	2	0	0	0	0	Down's syn	0
1	n	3	0	1	3	1	death, CAD	1
1	n	2	0	0	0	0		1
1	n	2	0	0	0	0		0
1	n	2	0	1	3	1	CAD and CA	0
1	n	2	0	1	3	1	h/o of CAB	1
1	n	1	1	0	0	0		0
1	n	1	1	0	0	0		0
1	n	2	0	1	3	1	h/o cardiac	1
1	n	1	1	0	0	0		0
1	y	1	1	0	0	0		0
1	n	2	0	0	0	0		0
1	n	1	1	0	0	0		0
1	n	3	0	1	0	0	PVD	0
1	n	1	1	0	0	0		0
1	n	2	0	0	1	0	1 DSE --> m	0
1	n	1	1	0	0	0		0
1	n	2	0	0	0	0		0
1	n	2	0	1	0	0	AF with RV	1
1	n	2	0	1	0	0	CHF in 199	0
1	n	1	1	0	0	0		0
1	n	2	0	1	0	0	h/o of pulm	0
1	n	2	0	0	0	0		0
1	n	2	0	0	0	0		0
1	n	1	1	0	0	0		0
1	n	2	0	1	3	1	CABG 2001	0
1	n	1	1	1	0	0	TIAs	0
1	n	1	1	1	3	1	CABG, PTCA	1
1	n	2	0	0	0	0		0
1	n	2	0	0	0	0		0
1	n	1	1	0	0	0		0
1	n	2	0	0	1	0	mild CAD n	0
1	y	1	1	0	0	0		0

1	n	2	0	0	0	0		0
1	n	1	1	0	0	0		0
1	n	2	0	1	0	0	h/o of hosp	0
0	n	4	0	0	0	0		0
1	n	3	0	0	0	0	withdrew f	0
1	n	1	1	0	0	0		0
1	n	3	0	1	3	1	CAD and DI	1
1	n	2	0	0	0	0		0
1	n	2	0	0	0	0		0
1	n	3	0	1	0	0	h/o of repe	0
0	n	1	1	0	0	0		0
0	n	4	0	1	3	1	PTCA	1
1	n	2	0	1	3	1	PTCA2013	0
1	n	1	1	0	0	0		0
1	n	1	1	1	1	0	CAD on ang	0
1	n	1	1	1	3	1	h/o of CAB	0
1	n	2	0	1	3	1	CABG 2012	0
1	n	2	0	0	0	0		0
1	n	1	1	0	0	0		0
0	n	4	0	1	0	0	CHF	0
1	n	2	0	1	0	0	PVD	0
1	n	1	1	1	3	1	PTCA 2012	0
1	n	2	0	1	3	1	CHF, CVA, A	0
1		3	0	0	0	0	death after	0
1	n	1	1	0	0	0		0
1	n	1	1	0	0	0		0
1	n	1	1	1	3	1	CAD, CABG	0
1	n	1	1	1	3	1	h/o CABG 2	1
1	n	1	1	0	0	0		0
1	n	1	1	0	0	0		0
1	n	2	0	0	0	0		0
1	n	2	0	0	0	0		0
1	n	2	0	0	0	0		0
1	n	2	0	0	0	0	nicotine de	0
1	n	2	0	0	0	0		0
1	n	2	0	0	0	0		0
1	n	5	0	0	0	0		0
0	n	4	0	1	3	1	CAD and CA	0
1	n	1	1	0	0	0		0
3	n	6	0	0	0	0		0
1	n	1	1	0	0	0		0
1	n	2	0	1	3	1	CAD and AI	0
1	n	2	0	0	0	0		0
1	n	1	1	1	0	0	PVD amput	1
1	n	1	1	1	1	0	HF and mil	0
1	n	2	0	1	3	1	PTCA after	1
3	n	6	0	0	1	0	mild CAD o	0

1	n	3	0	0	0	0	death	0
1	n	1	1	0	0	0		0
1	n	2	0	1	1	0	LVAD 2015	1
1	n	2	0	0	0	0		0
1	n	1	1	0	0	0	h/o atrial fl	0
1	n	2	0	0	0	0		0
3	n	6	0	1	0	0	CHF, PVD, I	1
1	n	1	1	0	0	0	severe COF	0
1	n	2	0	0	0	0	chronic lith	0
1	n	2	0	0	0	0		0
1	n	1	1	0	0	0		0
1	n	1	1	0	0	0		0
1	n	2	0	0	0	0		0
1	n	1	1	0	0	0		0
1	n	2	0	0	0	0		0
0	n	4	0	0	0	0		0
1	n	1	1	0	0	0		0
1	n	1	1	1	3	1	PTCA 04/2(	1
0	n	4	0	1	0	0	hypertensiv	0
1	n	2	0	0	0	0		0
1	n	1	1	0	0	0		0
1	n	1	1	1	0	0	CVA	0
1	n	1	1	0	0	0		0
1	n	2	0	0	0	0	started HD	0
0	n	4	0	0	0	0	got off dial	0
1	n	2	0	0	0	0		0
1	n	1	1	0	0	0		0
1	n	1	1	0	0	0		0
1	n	2	0	1	0	0		0
1	n	1	1	1	0	0		0
1	n	2	0	1	0	0		0
1	n	1	1	0	0	0		0
1	n	1	1	0	0	0		0
1	y	1	1	0	0	0		0
1	n	1	1	0	0	0		1
1	y	1	1	0	0	0		0
1	n	2	0	1	3	1	CAD with A	0
3	y	1	1	0	0	0		0
1	n	1	1	0	0	0		1
1	n	3	0	0	0	0		0
0	n	4	0	0	0	0		0
1	y	1	1	0	0	0		0
1	n	2	0	0	0	0		0
1	n	3	0	1	3	1		1
0	n	6	0	1	0	0	CVA	0
1	n	1	1	0	0	0		0
1	y	1	1	0	0	0		0

1	n	1	1	0	0	0		0
3	n	2	0	1	3	1	PCI x2 and	0
1	n	1	1	1	3	1	CAD with A	0
1	n	1	1	1	3	1	CABG	0
1	n	2	0	0	0	0		0
1	n	1	1	0	0	0		0
1	n	1	1	0	0	0		0
1	n	2	0	0	0	0		0
3	n	6	0	0	1	0		0
3	n	6	0	0	0	0	LVOT obstr	0
1	n	1	1	0	0	0		0
3	n	6	0	0	0	0		0
1	n	1	1	1	0	0	CVA	0
1	n	1	1	0	0	0		0
1	n	2	0	0	0	0		0
3	n	3	0	1	0	0	CVA	0
1	n	1	1	0	0	0		0
1	n	2	0	1	3	1	DES in 2014	1
0	n	3	0	1	0	0	death, CVA	0
1	n	1	1	0	0	0		0
0	n	4	0	0	0	0	h/o hypert	0
1	n	2	0	0	0	0		0
1	n	2	0	1	3	1	MI, DES 20	1
	n	5	0	0	0	0		0
1	n	2	0	0	0	0		0
1	n	1	1	1	1	0	CAD PTCA a	0
0	n	4	0	0	0	0		0
1	n	2	0	0	0	0	had severe	0
1	n	1	1	0	0	0		0
0	n	4	0	1	3	1	MI and ster	0
1	n	1	1	0	0	0		1
1	n	2	0	0	0	0		0
0	n	1	1	1	3	1	CVA and PT	1
1	n	2	0	0	0	0		0
0	n	4	0	1	3	1	CAD with P	0
1	n	1	1	0	0	0		0
1	n	1	1	0	0	0		0
0	n	4	0	1	0	0		1
1	n	1	1	0	0	0		0
1	n	2	0	0	0	0		0
1	n	4	0	1	0	0		0
1	n	1	1	0	0	0		0
1	n	1	1	0	0	0		0
1	n	1	1	0	0	0		0
1	n	2	0	1	3	1	PTCA at tim	1
3	n	6	0	1	0	0	PVD	0
1	n	2	0	0	0	0		0

1	n	1	1	0	0	0		0
3	n	6	0	0	0	0		0
1	n	2	0	0	0	0		0
1	n	2	0	0	0	0		0
1	n	2	0	0	0	0		0
1	n	2	0	1	3	1	AMI	0
1	n	2	0	1	3	1	CABG	0
1	n	2	0	0	0	0		0
1	n	1	1	0	0	0		0
1	n	2	0	0	0	0		0
3	n	2	0	1	3	1		0
3	n	6	0	0	0	0		0
1	n	2	0	0	0	0	hypertroph	0

CVE post+D	Any cardiac	Column	death	MCVE	death AND	time to dea	censor	time to last
0		28.2	0	0	0		1	5
0	flash pul ec	25	0	0	0		1	12
1	high outpu	38.6	0	0	0	8	0	8
1		42.4	1	0	0	22	0	22
0	CAD, not ar	33.8	0	1	0	26	1	26
0		11.8	0	0	0		1	13
1	abnormal e	45.4	1	1	0	15	0	1
1	renal artery	13.7	0	0	0	16	0	16
0		15.8	0	0	0		1	11
0		32.2	0	0	0		1	33
0		31.8	0	0	0		1	27
1	CVA	34.4	0	1	0	0	0	0
0		27	0	0	0		1	9
0		46.7	0	0	0		1	27
1	PVD and isc	34	1	1	0	29	0	29
1	PTCA and D	13.6	0	0	0	17	0	17
0		14.7	0	0	0		1	28
0		26.1	0	1	0		1	33
1	positive DS	31.2	0	1	0	0	0	32
0		25.9	0	0	0		1	7
0	A flutter wi	17.5	0	0	0		1	21
1	CABGx1	47.9	0	1	0	7	0	7
0		14.6	0	0	0		1	3
0		22	0	0	0		1	7
0		29.7	0	0	0		1	34
0	mild CAD, (	45.7	0	0	0		1	19
1		33.1	1	1	0	2	0	32
0		19.3	0	0	0		1	17
0	mild CAD, (	29.6	0	0	0		1	30
0		17.4	0	0	0		1	29
0		16	0	0	0		1	22
1	Af induced	42.9	0	1	0	0	0	0
0		16	0	1	0		1	30
0		23.6	0	0	0		1	6
0		36.6	0	1	0		1	30
0		32	0	0	0		1	28
0		16.5	0	0	0		1	29
0		43.9	0	0	0		1	3
0		18	0	1	0		1	24
0		18.7	0	1	0		1	23
1	CABG x3	50.3	0	1	0	0	0	11
0		12.6	0	0	0		1	28
0		14.9	0	0	0		1	28
0		15.8	0	0	0		1	4
0	mild CAD o	31	0	1	0		1	27
0		24.8	0	0	0		1	11

0		29.2	0	1	0		1	27
0		5.9	0	0	0		1	0
0		47.2	0	1	0		1	25
0		34.7	0	0	0		1	29
1		29	1	1	0	12	0	12
0		29.1	0	0	0		1	27
1		33.3	1	1	0	16	0	16
0		17.8	0	0	0		1	24
0		44.8	0	0	0		1	26
1		27.7	1	1	0	7	0	7
0		26.2	0	0	0		1	3
1	CAD and PT	365	0	1	0	7	0	25
0		50.1	0	0	0		1	23
0		40.8	0	0	0		1	17
0		33.8	0	1	0		1	0
0		13.6	0	1	0		1	26
0		6.8	0	1	0		1	24
0		21.6	0	0	0		1	27
0		75.7	0	0	0		1	3
0		46.4	0	1	0		1	27
0		56.3	0	1	0		1	28
0		14	0	1	0		1	6
0		55.3	0	1	0		1	24
1		20.3	1	0	0	5	0	5
0		62.7	0	0	0		1	8
0		18.4	0	0	0		1	7
0		34	0	1	0		1	16
1	CABG x4	20.5	0	1	0	1	0	1
0		5.1	0	0	0		1	10
0	AF with RV	6.3	0	0	0		1	3
0		15.2	0	0	0		1	23
0		34.6	0	0	0		1	26
0		48.1	0	0	0		1	26
0		16.5	0	0	0		1	26
0		20.4	0	0	0		1	26
0		10.4	0	0	0		1	26
0		43.4	0	0	0		1	26
0		33.1	0	1	0		1	26
0		19.3	0	0	0		1	4
0		40.1	0	0	0		1	27
0		16.1	0	0	0		1	4
0		30.5	0	1	0		1	27
0		55.9	0	0	0		1	24
1	PVD s/p am	15.3	0	1	0	22	0	22
0	occluded v	40.7	0	1	0	4	1	13
1	CAD s/p DE	33.9	0	1	0	0	0	24
1		152.5	1	0	0	6	0	24

1		19.3	1	0	0	13	0	25
0		13.4	0	0	0		1	3
1	LVAD for e	16.5	0	1	0	19	0	23
0		19.3	0	0	0		1	27
0	apical hype	28	0	0	0		1	3
0		25.4	0	0	0		1	31
1	new worse	51	0	1	0	30	0	30
0		37.8	0	0	0		1	11
0		36.5	0	0	0		1	31
0		44.2	0	0	0		1	29
0		22.2	0	0	0		1	3
0		22.2	0	0	0		1	8
0		27.7	0	0	0		1	34
0		23.6	0	0	0		1	13
0		22.1	0	0	0		1	33
0		27.8	0	0	0		1	32
0		31.5	0	0	0		1	4
1	CAD s/p PT	29.1	0	1	0	1	0	1
0		46.2	0	1	0	0	1	30
0		21.6	0	0	0		1	30
0		37.6	0	0	0		1	23
0		30.3	0	1	0		1	24
0		28.4	0	0	0		1	8
0		21.9	0	0	0		1	33
0		33.4	0	0	0		1	33
0		24.3	0	0	0		1	33
0		22.7	0	0	0		1	30
0		36.5	0	0	0		1	25
0		42.3	0	1	0		1	32
0		39.8	0	1	0		1	2
0		55.4	0	1	0		1	32
0		26.3	0	0	0		1	4
0		23.8	0	0	0		1	5
0		29.9	0	0	0		1	4
1	biventricul	15.7	0	0	0	3	0	3
0		33.8	0	0	0		1	20
0		39.3	0	1	0		1	33
0		29.9	0	0	0		1	21
1	CABG x2	26.4	0	0	0	19	0	19
1		161.1	1	0	0	2	0	2
0		26.5	0	0	0		1	32
0		18.4	0	0	0		1	22
0		21.3	0	0	0		1	32
1	PTCA and D	41.4	1	1	0	20	0	20
0		26.1	0	1	0		1	34
0		14.2	0	0	0		1	3
0		69	0	0	0		1	11

0		15.9	0	0	0		1	23
0		27.4	0	1	0		1	25
0		37.8	0	1	0		1	5
0		31.6	0	1	0		1	10
0		25.9	0	0	0		1	24
0		43.9	0	0	0		1	2
0		24.1	0	0	0		1	11
0		18.4	0	0	0		1	25
0	AF 081420:	20.65	0	0	0		1	25
0		89.5	0	0	0		1	25
0		18.7	0	0	0		1	4
0		48.8	0	0	0		1	24
0		24.6	0	1	0		1	4
0		25.8	0	0	0		1	25
0		13.3	0	0	0		1	30
1		32.9	1	1	0	3	0	3
0		23.2	0	0	0		1	7
1	CAD s/p DE	17.3	0	1	0	20	0	20
1		34.1	1	1	0	25	0	25
0		26.2	0	0	0		1	20
0		13.6	0	0	0		1	29
0		48.8	0	0	0		1	29
1	CAD sp DES	29.1	0	1	0	18	0	18
0		22.9	0	0	0		1	20
0		37.3	0	0	0		1	28
0		27.8	0	1	0		1	7
0		53.3	0	0	0		1	28
0		154.4	0	0	0		1	28
0		14.3	0	0	0		1	17
0		25.1	0	1	0		1	25
1	CAD sp DES	21.4	0	0	0	24	0	24
0		36.8	0	0	0		1	31
1	CAD s/p DE	168.6	0	1	0	18	0	18
0		18.7	0	0	0		1	29
0	moderate (	20.2	0	1	0		1	28
0		9.7	0	0	0		1	18
0		28.4	0	0	0		1	3
1	PVD s/p an	32.3	0	1	0	25	0	25
0		13.3	0	0	0		1	5
0		36.4	0	0	0		1	28
0	diagnosed	18.7	0	1	0		1	28
0		350	0	0	0		1	3
0		49.6	0	0	0		1	15
0		10.7	0	0	0		1	3
1	CAD s/p PT	21.2	0	1	0	0	0	0
1		93.7	1	1	0	9	0	9
0		14.9	0	0	0		1	27

0	25.5	0	0	0	1	2
0	22.1	0	0	0	1	28
0	22.7	0	0	0	1	23
0	19	0	0	0	1	28
0	32.2	0	0	0	1	29
0	23.5	0	1	0	1	26
0	23.3	0	1	0	1	34
0	31.5	0	0	0	1	26
0	46.7	0	0	0	1	18
0	50	0	0	0	1	28
0	27.9	0	1	0	1	27
0	102.5	0	0	0	1	27
0	32.6	0	0	0	1	25

: f/u or time to transplant or time to event
